# Embedding of Markov matrices for $\varvec{d \leqslant 4}$

**DOI:** 10.1007/s00285-024-02112-w

**Published:** 2024-07-02

**Authors:** Michael Baake, Jeremy Sumner

**Affiliations:** 1 Fakultät für Mathematik, Universität Bielefeld, Postfach 100131, 33501 Bielefeld, Germany; 2 School of Natural Sciences, Discipline of Mathematics, University of Tasmania, Private Bag 37, Hobart, TAS 7001 Australia

**Keywords:** Markov matrices and generators, Embedding problem, Phylogenetics, 60J10, 60J27, 92D10

## Abstract

The embedding problem of Markov matrices in Markov semigroups is a classic problem that regained a lot of impetus and activities through recent needs in phylogeny and population genetics. Here, we give an account for dimensions $d\leqslant 4$, including a complete and simplified treatment of the case $d=3$, and derive the results in a systematic fashion, with an eye on the potential applications. Further, we reconsider the setup of the corresponding problem for time-inhomogeneous Markov chains, which is needed for real-world applications because transition rates need not be constant over time. Additional cases of this more general embedding occur for any $d\geqslant 3$. We review the known case of $d=3$ and describe the setting for future work on $d=4$.

## Introduction

Markov models are the backbone of many if not most stochastic processes. In real-world applications, they usually appear with finite state spaces, and come in two flavours, namely with discrete or with continuous time. While discrete-time Markov chains often appear simpler on first inspection, they are connected to their continuous-time counterparts via the obvious question when they admit a continuous interpolation. This was identified as an important problem by Elfving (1937) because pure discrete-time models may have some problems or even inconsistencies for the intended application. Clearly, this question is also relevant in biological models, for instance in phylogeny and population genetics. Indeed, when modelling the time evolution of genetic sequences built from the nucleotide alphabet $\{A, G, C, T\}$, one is right in the middle of this type of interpolation problem. It can essentially be rephrased as asking whether a given Markov matrix can be embedded into a Markov semigroup or, more generally, into a Markov flow, thus referring to underlying continuous-time processes that are homogeneous or non-homogeneous in time, respectively. The purpose of this paper is to provide methods and results to answer the embedding problem for this type of application.

Let us be a bit more specific. Phylogenetic models typically make the strong assumption that random changes in nucleotide sequences follow a Markov process that is both stationary (in nucleotide composition) and homogeneous (meaning that instantaneous substitution rates are constant in time and across biological lineages). However, there is abundant biological evidence that the molecular evolution of certain groupings of organisms does not strictly adhere to the typical assumption of a stationary and homogeneous Markov process; for example, Cox et al. (2008) establish significant nucleotide compositional differences across the tree of life, while Jayaswal et al. (2014) establish the same for a yeast phylogeny. For this reason, there are various studies that assess the possibility of fitting non-stationary models of molecular evolution; compare (Jayaswal et al. 2011). The embedding problem therefore has important practical application in phylogenetics as its solutions allow (at least theoretically) for the detection of non-homogeneous Markov processes in historical molecular evolution.

Let us now describe our setting from a more mathematical perspective. A *Markov matrix* *M* is a non-negative square matrix with all row sums equal to one, which is the convention we use here. The set of all Markov matrices with *d* states (or in *d* dimensions) is denoted by $\mathcal {M}_d$, which is a closed convex subset of the real $d {\times } d$-matrices. A *rate matrix* *Q* is a real matrix with non-negative off-diagonal entries and all row sums equal to zero. Such matrices are also known as *Markov generators*, or simply *generators*, due to their connection with (time-homogeneous) Markov semigroups, as given by $\{ \textrm{e}^{t Q}: t \geqslant 0 \}$, which is a monoid of Markov matrices.

It is an old question, known as Elfving’s interpolation problem (Elfving 1937), whether a given Markov matrix *M* can occur within a Markov semigroup. This is equivalent to asking whether there is a generator *Q* such that $M=\textrm{e}^Q$, which simply sets a particular time scale. We call this the (classic) *embedding problem*. It became famous through a foundational paper by Kingman (1962), which also included a simple criterion for $d=2$ due to Kendall and attracted a lot of research for many years; see Davies (2010), Baake and Sumner (2020) and references therein. Several general characterisations were found, while concrete, applicable criteria in higher dimensions turned out to be more difficult. After settling $d=3$, see Davies (2010), Baake and Sumner (2020) and references therein for an account of the history, and several papers in mathematical finance, sociology and statistics, compare (Higham 2008, Sec. 2.3), the interest in the problem faded somewhat, as no driving application was in sight. In particular, no systematic treatment of $d>3$ was done. A similar fate was met by the generalised embedding problem of a given Markov matrix in a time-inhomogeneous process (Frydman and Singer 1979; Johansen and Ramsey 1979). Also, it was clear that, in higher dimensions, the embedding problem will become increasingly more complex, due to topological properties already discussed in Kingman (1962) and due to the multiple possibilities for degenerate spectra with repeated Jordan blocks. To some extent, this starts in $d=4$, and no impetus was visible to work on a classification.

This changed through the rise of bioinformatics, which needed the case $d=4$ solved explicitly, with effective and concrete criteria, due to the importance of various Markov processes on the genetic (or nucleotide) alphabet $\{ A, G, C, T \}$. Here, processes of molecular evolution became relevant in phylogenetics, as one typical problem is the inference of continuous-time processes such as mutation or recombination from discrete data sets. The transition rates of real-world Markov processes should be expected to change over time (Cox et al. 2008). For this and various other reasons, discrete-time models that are not embeddable in any reasonable way appear, in our opinion, questionable or too restrictive for applications to overlapping generations or to other processes that require continuous time. In particular, this is certainly a natural point of view for processes over a long time period, as one encounters in phylogenetics. Recently, the embedding problem for $d=4$ was largely solved in Casanellas et al. (2023) via a detailed case distinction on the basis of the possible Jordan normal forms, including algorithms to decide some of the more complicated cases. One small disadvantage of this approach is some dependence on the similarity transform between the matrix and its *Jordan normal form* (JNF).

Here, we take a different, more algebraic path to the problem for $d\leqslant 4$ that is based on the degree of the minimal polynomial of the matrix. While some cases will still require the JNF, the treatment of cyclic matrices (which are those where minimal and characteristic polynomial agree and are thus generic in a measure-theoretic sense—to be made more precise below) will become more systematic, and the emerging embedding criteria are explicit and easy to implement. The golden key in this generic case, as well as in several of the other ones, is the proper use of the *spectral mapping theorem* (SMT), see Hille and Phillips (1957, Sec. 5.3) for the general setting and Eq. (3) below for a formulation, in conjunction with Frobenius’ theorem on the centraliser of a cyclic matrix, as developed in some detail in Baake and Sumner (2020); see Theorem 2.1 for specific details. In most cases, this leads to explicitly solving a simple set of linear equations and checking whether the solution is a generator or not.

Then, we look at the corresponding problem with time-dependent generators. While this does not give new cases for $d=2$, things change already for $d=3$, as has been known since the 1970s (Goodman 1970; Johansen 1973). Consequently, embeddability in this generalised sense is relevant, also in view of the applications. Fortunately, quite a bit of literature exists already, and a powerful connection with a problem from control theory (Frydman and Singer 1979; Johansen and Ramsey 1979; Frydman 1980a, b, 1983) makes some concrete results possible, though mainly for $d=3$. Here, we review and extend these results in our setting.

The paper is organised as follows. After some recollections of general results in Sect. 2, which includes the complete answer for $d=2$, we discuss the case $d=3$ in Sect. 3. While this is classic material, we are not aware of a systematic presentation in one place, and this simultaneously allows us to introduce further methods and tools as we go along. In this sense, our paper is both a partial review and an extension of known results at the same time. In particular, we take a fresh look at the most difficult case, which consists of the diagonalisable Markov matrices with a negative eigenvalue of multiplicity 2. Here, we give a complete solution that is both simple and constructive. A summary is provided in Table 1.

In our exposition, for the sake of better readability, we often give the arguments first and only then state the result, thus deviating from a purely formal presentation. In this context, in line with many other mathematical texts of a more expository nature, we use the symbol $\Box$ to denote the end or the *absence* of a proof, the latter when we cite a result from another source or when the arguments for the proof were given prior to the statement of the result.

Section 4 then deals with $d=4$, in the same spirit, where we spend some time to spell out the cyclic cases; a summary is given in Table 2. This is followed by applications to some important examples from phylogenetics in Sect. 5, which, together with the progress in Casanellas et al. (2023), motivated the entire endeavour in the first place. Finally, in Sect. 6, we summarise the embedding problem for time-inhomogeneous Markov chains, where we can restrict our main attention to piecewise continuous families of generators without loss of generality, as a result of the Bang–Bang principle from control theory that applies in this setting (Johansen 1973; Johansen and Ramsey 1979; Frydman 1980b). Here, we set the scene for future work, in particular on $d=4$.

## Preliminaries and general background results

Let us recall some notation and results on matrices that we will employ throughout, mainly following (Gantmacher 1986; Horn and Johnson 2013; Baake and Sumner 2020, 2022). We use $\textrm{Mat}(d,\mathbb {R})$ and $\textrm{Mat}(d,\mathbb {C})$ for the rings of $d{\times } d$-matrices over the real and the complex numbers, respectively. We use  to denote the unit matrix, where we will simply write  whenever the dimension is clear from the context. Further, we use

1 $$\begin{aligned} \mathcal {A}^{(d)}_{0} :=\{ A \in \textrm{Mat}(d,\mathbb {R}): \text {all row sums are}\,0 \} , \end{aligned}$$

which is a non-unital algebra of dimension $d(d{-}1)$. Given some $B\in \textrm{Mat}(d,\mathbb {C})$, its *spectrum* is the set of eigenvalues of *B*, denoted by $\sigma (B)$ and primarily viewed as a set (and not as a multi-set, unless stated otherwise). The *characteristic polynomial* of *B* is2where $\mathfrak {m}_{\textsf{a}}(\lambda )$ is the *algebraic* multiplicity of $\lambda$, so $\sum _{\lambda \in \sigma (B)} \mathfrak {m}_{\textsf{a}}(\lambda ) = d$, while the *geometric* multiplicity is $\mathfrak {m}_{\textsf{g}}(\lambda ) = \dim (V_{\lambda })$, with $V_{\lambda } = \{ x \in \mathbb {C}^d: B x = \lambda x \}$ denoting the eigenspace for $\lambda \in \sigma (B)$ as usual. When *B* is a matrix and *p*(*x*) is any polynomial in one variable, also *p*(*B*) is well defined, and the SMT (Rudin 1991, Thm. 10.33) applied to matrices states that

3 $$\begin{aligned} \sigma \bigl ( p (B) \bigr ) \, = \, p \bigl ( \sigma (B) \bigr ) :=\{ p (\lambda ): \lambda \in \sigma (B) \} , \end{aligned}$$

which holds for the spectrum including multiplicities. In this context, the Cayley–Hamilton theorem (Horn and Johnson 2013, Thm. 2.4.3.2) asserts that , where we use  (or ) to denote the all-zero matrix.

We shall also need the *minimal polynomial* of *B*, called $q_{_B}$, which is the minimal monic factor of $p_{_B}$ that still satisfies . It is given by

4 $$\begin{aligned} q_{_B} (x) = \prod _{\lambda \in \sigma (B)} (x-\lambda )^{r_{max^{(\lambda )}}}, \end{aligned}$$

where $r_{max^{(\lambda )}}$ is the largest dimension of the elementary Jordan blocks for the eigenvalue $\lambda$. Note that we have dropped the factor $(-1)^d$ to make it monic (leading coefficient is 1), which is common, but not always done.

A matrix $B\in \textrm{Mat}(d, \mathbb {C})$ is called *simple* when its eigenvalues are distinct, which is to say that $\mathfrak {m}_{\textsf{a}}(\lambda ) = \mathfrak {m}_{\textsf{g}}(\lambda ) = 1$ for all $\lambda \in \sigma (B)$, and *degenerate* otherwise. Further, *B* is called *cyclic* when $q_{_B} = (-1)^d \, p_{_B}$, that is, when characteristic and minimal polynomial agree (possibly up to a sign). Cyclic matrices are the ones with $\mathfrak {m}_{\textsf{g}}(\lambda ) = 1$ for all $\lambda \in \sigma (B)$, which means that the corresponding Jordan blocks in the standard (complex) JNF of *B* are $\mathbb {J}_{\mathfrak {m}_{\textsf{a}}(\lambda )} (\lambda )$. Here, , with $N_n \in \textrm{Mat}(n,\mathbb {R})$ denoting the matrix with 1s on the first super-diagonal and $0$s everywhere else. A matrix $\mathbb {J}_n (\lambda )$ is called an *elementary Jordan block*. $\mathbb {J}_{1} (\lambda )$, which is still diagonal, is called *trivial*, while all $\mathbb {J}_n (\lambda )$ with $n\geqslant 2$ are *non-trivial*. Clearly, $N_n$ is *nilpotent*, with  for all $m\geqslant n$, but , where *n* is called the *degree* of $N_n$.

In particular, simple matrices are cyclic, but sometimes too restrictive (though still generic). Here and below, we use the word *generic* for an attribute in a measure-theoretic sense, thus referring to the property that the objects without this attribute form a null set. Further, the use of cyclic matrices is natural due to Frobenius’ theorem, which we recall as follows for the case of real matrices; see Baake and Sumner (2020, Fact 2.10) and references given there for details. Here and below, we use $[A,B ] :=AB - BA$ for the (commutator) of two matrices.

### Theorem 2.1

For $B\in \textrm{Mat}(d, \mathbb {R})$, the following properties are equivalent.

1. *B* is *cyclic*, that is, the characteristic polynomial of *B* is, possibly up to a sign, also its minimal polynomial, so $q_{_B} = (-1)^d p_{_B}$ with $p_{_B}$ and $q_{_B}$ from Eqs. (2) and (4).
2. There is a vector $v\in \mathbb {R}^d$ such that $\{ v, Bv, B^2v, \ldots , B^{d-1}v \}$ is a basis of $\mathbb {R}^d$.
3. *B* is *non-derogatory* (Horn and Johnson 2013), that is, one has $\mathfrak {m}_{\textsf{g}}(\lambda ) = 1$ for all $\lambda \in \sigma (B)$.
4. For each $\lambda \in \sigma (B)$, the corresponding Jordan block of the JNF is $\mathbb {J}_{\mathfrak {m}_{\textsf{a}}(\lambda )} (\lambda )$.
5. The centraliser of $B$, , is Abelian.
6. One has $\textrm{cent}(B) = \mathbb {R}[B]$, where $\mathbb {R}[B]$ is the polynomial ring generated by *B*. $\square$

The second property explains the name, and holds in this way because eigenvalues and eigenvectors, as well as principal vectors, are real or come in complex-conjugate pairs. The last property in Theorem 2.1 is the most powerful in our context, because one hasby standard arguments. Property (6) is equivalent to saying that every matrix that commutes with the cyclic matrix *B* is a polynomial in *B* with real coefficients and degree at most $d-1$; see Horn and Johnson (2013, Thm. 3.2.4.2) for details. We shall make use of this relation many times.

### Fact 2.2

For all $M\in \mathcal {M}_d$, one has $1\in \sigma (M)$ together with $\mathfrak {m}_{\textsf{g}}(1) = \mathfrak {m}_{\textsf{a}}(1)$. In particular, there is no non-trivial Jordan block for $\lambda = 1$.

Further, the corresponding statement holds for generators, which is to say that any generator has 0 as an eigenvalue, with equal algebraic and geometric multiplicity.

### Proof

The statement for *M* is standard; see Gantmacher (1986, Thm. 13.10) or Baake and Sumner (2020, Prop. 2.3(2)).

If *Q* is an arbitrary generator, one can always find a number $a > 0$ such that  is Markov, from which the claim on generators is immediate. $\square$

For a given matrix *B*, its exponential is defined by the standard power series $\textrm{e}^B = \sum _{n=1}^{\infty } \frac{1}{n!}B^{n}$, which always converges; see Higham (2008, Ch. 10) for details. Due to the Cayley–Hamilton theorem, it can be expressed as a polynomial in *B* as well, and one always has . Further, we have the following property, which we will need repeatedly.

### Fact 2.3

(Baake and Sumner 2020, Fact 2.15) Let $B\in \textrm{Mat}(d,\mathbb {C})$. If $\textrm{e}^B$ is diagonalisable, then so is *B*. $\square$

When $d=1$, the only Markov ‘matrix’ is $M=1$, and the only ‘generator’ is $Q=0$. They connect via $1 = \textrm{e}^0$, which is the unique relation here. For this reason, nothing of interest happens in one dimension, and we shall generally assume $d\geqslant 2$ to avoid trivialities.

For $d=2$, one has

$$\begin{aligned} \mathcal {M}^{}_{2} = \left\{ \left( \begin{array}{cc} 1{-}a &{} a \\ b &{} 1{-}b \end{array} \right) : a,b \in [0,1] \right\} , \end{aligned}$$

and the embedding problem is completely solved by Kendall’s theorem, which was not published by himself; see Kingman (1962, Prop. 2) or Baake and Sumner (2020, Thm. 1) for accounts with proofs.

### Theorem 2.4

(Kendall) The Markov matrix $$M = \left( {\begin{matrix} 1-a &{} a \\ b &{} 1-b \end{matrix}} \right) $$ with $a,b\in [ 0,1]$ is embeddable if and only if $\det (M) > 0$, which is also equivalent to the condition $0 \leqslant a+b <1$. In this case, there is precisely one generator *Q* such that $M=\textrm{e}^Q$, namely . $\square$

Here, we have the best possible situation, in that there is a simple necessary and sufficient criterion, and, in the affirmative case, a closed formula for the unique generator. The reason for its simplicity has to do with the fact that all  are simple (hence also cyclic) and have real spectrum. Then, embeddability occurs if and only if *M* has positive spectrum, which means $\sigma (M) \subset \mathbb {R}_{+}$; compare (Baake and Sumner 2020). Parts of this structure are still present when one looks into particular classes of Markov matrices for $d>2$, but not in general. In fact, much of this paper is concerned with the complications that emerge for $d=3$ and $d=4$. General results for $d>4$ only exist for special types of matrices; see the discussion in Higham (2008) and Baake and Sumner (2022) for details.

In particular, when $M\in \mathcal {M}_d$ is cyclic and has positive spectrum, the derived rate matrix  has spectral radius $\varrho _{_A} <1$. Then, the principal logarithm via the power series5converges in norm, and defines a real matrix with zero row sums. Note that the actual calculation of  usually employs the minimal polynomial of *A*, by which the logarithm can be expressed as a polynomial in *A*; compare (Higham 2008; Baake and Sumner 2022). Now, one has the following general result (Baake and Sumner 2022, Thm. 5.3 and Cor. 5.5) on (classic) embeddability; see also Higham (2008, p. 38).

### Theorem 2.5

Let $M\in \mathcal {M}_d$ be cyclic, with real spectrum. Then, *M* is embeddable if and only if the following two conditions are satisfied, where .

1. The spectrum of *M* is positive, that is, $\sigma (M) \subset \mathbb {R}_{+}$.
2. The matrix  is a generator.

In this case, *Q* is the principal matrix logarithm of *M*, and the embedding is unique, even in the sense that there is no other real logarithm of *M* with zero row sums. $\square$

The term *real logarithm* of *M* refers to any matrix $R\in \textrm{Mat}(d,\mathbb {R})$ with $\textrm{e}^R = M$. The following important characterisation follows from Culver (1966, Thms. 1 and 2), which is a refinement of classic results from Gantmacher (1986, Ch. 5).

### Fact 2.6

A matrix $B\in \textrm{Mat}(d,\mathbb {R})$ has a real logarithm if and only if the following two conditions are satisfied.

1. The matrix *B* is non-singular.
2. Each elementary Jordan block of the JNF of *B* that belongs to a negative eigenvalue occurs with even multiplicity.

Further, when all eigenvalues of *B* are positive real numbers and no elementary Jordan block occurs twice, the real logarithm of *B* is unique. $\square$

Below, we refer to the existence part of Fact 2.6 as *Culver’s criterion*. Note that, for the uniqueness statement, the matrix *B* need not be cyclic, as it can have two different Jordan blocks for the same (positive) eigenvalue, and still satisfy the condition.

### Remark 2.7

When Culver’s criterion for negative eigenvalues is satisfied, or when any elementary Jordan block of a positive real eigenvalue of *B* occurs more than once, there are *uncountably* many real logarithms. To see this, observe that one can start from one real logarithm with the matching JNF, and modify a pair of blocks by adding $2 \pi \textrm{i}k$ to the eigenvalue of one and its complex conjugate to the eigenvalue of the other, for some $0\ne k\in \mathbb {Z}$. This will not change the exponential of the matrix, but its symmetry. Indeed, while any matrix in $\textrm{GL}(2,\mathbb {R})$ commutes with , they need not (and generally will not) commute with $\textrm{diag}(\lambda +2 \pi \textrm{i}k, \lambda - 2 \pi \textrm{i}k)$ when $k\ne 0$, and similarly for block matrices with two equal versus two modified Jordan blocks. We will meet one instance of this in Lemma 2.12.

The other possible mechanism occurs if *B* has a pair of complex conjugate eigenvalues, and is then a direct consequence of the structure of the complex logarithm. This results in a *countable* set of real logarithms. These are the two possible mechanisms for additional real logarithms, both of which will show up below; see Higham (2008) or the discussion around the Corollary in Culver (1966) for details. It will be our task to identify the generators among them. $\Diamond$

Before we continue, let us formulate one simple necessary criterion for embeddability due to Elfving (1937), and its consequence on the spectrum of an embeddable matrix.

### Fact 2.8

If $M\in \mathcal {M}_d$ is embeddable, no diagonal element of *M* can be zero. Further, $\lambda =1$ is the only eigenvalue of *M* on the unit circle, and all other eigenvalues satisfy $|\lambda |< 1$.

### Proof

Let $M=\textrm{e}^Q$ with a generator *Q*, and consider $M(t)= \textrm{e}^{t Q}$ for $t\in [0,1]$, which is a continuous path in $\mathcal {M}_d$ from  to $M(1)=M$. By continuity, $M\bigl ( \frac{1}{n}\bigr )$ must have all diagonal elements strictly positive, for some sufficiently large $n\in \mathbb {N}$. Then, with $M(t) = \bigl ( m^{}_{ij} (t) \bigr )_{1\leqslant i,j \leqslant d}$, we get $m^{}_{ii} (1) \geqslant \bigl ( m^{}_{ii} (n^{-1})\bigr )^{n} > 0$ for all $1\leqslant i \leqslant d$ as claimed.

Clearly, all eigenvalues of a Markov matrix satisfy $|\lambda |\leqslant 1$ by the *Perron–Frobenius* (PF) theorem; see Horn and Johnson (2013, Sec. 8.3) for background. Now, if *M* is embeddable, all diagonal elements are strictly positive. Let *p* be the smallest of them, and consider , which is still a non-negative matrix, so the modulus of all of its eigenvalues is bounded by its PF eigenvalue, which gives $|\lambda - p |\leqslant 1-p$ for all $\lambda \in \sigma (M)$. So, $\sigma (M)$ is contained in a closed disk of radius $1-p$ that lies inside the unit disk in such a way that it touches 1, but no other point of the boundary, so $\lambda =1$ or $|\lambda |<1$. $\square$

Now, (classic) embeddability of $M \in \mathcal {M}_d$ means $M = \textrm{e}^Q$ for a generator *Q*, and the set of all embeddable Markov matrices, for fixed *d*, is denoted by $\mathcal {M}^{\textrm{e}}_{d}$. Clearly, embeddability is a special case of the existence of a real logarithm, because each generator is a real logarithm, while the converse is generally not true. This refers to the hard part of the embedding problem, namely establishing the required non-negativity conditions for the off-diagonal matrix elements of the logarithm. Let us begin with a simple result.

### Fact 2.9

The only generator $Q = (q^{}_{ij} )^{}_{1\leqslant i,j \leqslant d}$ that satisfies  is .

### Proof

The claim is trivial for $d=1$. Since , we get $\textrm{tr}(Q) = 0$ as *Q* is real. Now, the generator property of *Q* implies that all $q^{}_{ii} \leqslant 0$, so $\textrm{tr}(Q) = \sum _{i} q^{}_{ii}$ forces $0=q^{}_{ii} = -\sum _{i\ne j} q^{}_{ij}$ for all *i*, hence $q^{}_{ij} = 0$ for all $i\ne j$, and  as claimed. $\square$

Note that  has uncountably many real logarithms, though only one with zero row sums, the generator . As we shall need this later for several cases with degenerate spectrum, let us expand on this point a little. The equation $\textrm{e}^z = 1$ with $z\in \mathbb {C}$ holds if and only if $z = 2 \pi \textrm{i}k$ for some $k\in \mathbb {Z}$, and we need the analogue of this in the sense that we want all solutions of  with $R\in \textrm{Mat}(2,\mathbb {R})$. Such solutions exist, as one sees from  with $$I = \left( {\begin{matrix} 0 &{} -1 \\ 1 &{} 0 \end{matrix}}\right) $$, and  holds if and only if $\gamma = 2 \pi k$ for some $k\in \mathbb {Z}$, whereThe general result reads as follows.

### Fact 2.10

The real logarithms of  are precisely the matrices $2 \pi k I_{x,y,z}$ with

$$\begin{aligned} I_{x,y,z} :=\begin{pmatrix} x &{} -z \\ y &{} -x \end{pmatrix}, \end{aligned}$$

where $k\in \mathbb {N}_0$ and $x,y,z\in \mathbb {R}$ such that $yz-x^2=1$. The parametrisation is unique, and the only real logarithm with zero row sums is the one with $k=0$.

### Proof

The relation  forces *R* to be diagonalisable over $\mathbb {C}$ by Fact 2.3. As *R* should be real, it must then be similar to $2 \pi k \, \textrm{diag}(\textrm{i}, -\textrm{i})$ within $\textrm{GL}(2,\mathbb {C})$, for some $k\in \mathbb {Z}$. Writing $$B = \left( {\begin{matrix} a &{} b \\ c &{} d \end{matrix}}\right) $$ with complex entries and $\det (B) = ad-bc \ne 0$, a quick calculation shows that $B \, \textrm{diag}(\textrm{i}, - \textrm{i}) B^{-1}$, which always lies in $\textrm{SL} (2,\mathbb {C})$, must be of the form $$I_{x,y,z} = \left( {\begin{matrix} x &{} -z \\ y &{} -x \end{matrix}}\right) $$ with $x,y,z \in \mathbb {C}$ and $yz-x^2=1$. We get all *real* solutions by restricting the entries to $\mathbb {R}$.

By construction, these matrices are the most general real square roots of  in $\textrm{GL}(2,\mathbb {R})$, which can also be calculated directly. Consequently, we get  for all $k\in \mathbb {Z}$ and $x,y,z\in \mathbb {R}$ with $yz-x^2=1$. Since $I_{-x,-y,-z} = - I_{x,y,z}$ and since $I_{x,y,z}=I_{x',y',z'}$ holds only for $x=x'$, $y=y'$ and $z=z'$, the claim is clear; see (Casanellas et al. 2023) for a similar treatment. $\square$

Sometimes, it is advantageous to admit $k\in \mathbb {Z}$ and restrict *z* to be positive, which gives another unique parametrisation. Note that *y* must then be positive as well. We shall make use of this freedom without further notice. Every matrix $I_{x,y,z}$ from Fact 2.10 has eigenvalues $\pm \textrm{i}$ and is thus simple. Then, the commutation relation , as a consequence of Theorem 2.1(6), implies $(x',y',z') = \pm (x,y,z)$. Indeed, we have6This gives the following result.

### Corollary 2.11

Let $\lambda \ne 0$ be a fixed real number. Then, the most general real logarithms of  are the following matrices.

1. If .
2. If .

In both cases, the parametrisation is unique.

### Proof

Both claims follow from elementary calculations of the above type via (6). Since similar ones also appear in Casanellas et al. (2023), we leave the details to the interested reader. The only additional subtlety occurs for the second case, where one has to show that a single matrix $I_{x,y,z}$ suffices, which is a consequence of the commutation properties in (6). $\square$

Already for $d=3$, the situation ‘complexifies’, because  also has uncountably many real logarithms with zero row sums (Baake and Sumner 2022, Rem. 2.11). This is deeply connected with some of the difficulties of the embedding problem. However, the following variant of the uniqueness result in Fact 2.6 will become important below.

### Lemma 2.12

Let $M\in \mathcal {M}_d$ be a Markov matrix with the following properties.

1. All eigenvalues of *M* are positive, so $\sigma (M) \subset \mathbb {R}_{+}$.
2. For $1\ne \lambda \in \sigma (M)$, no elementary Jordan block in the JNF occurs twice.
3. The multiplicity of $\lambda = 1$ is $\mathfrak {m}_{\textsf{a}}(1) = 2$.

Then, there are uncountably many real logarithms, but only one of them lies in $\mathcal {A}^{(d)}_{0}$, which then is the only candidate for a generator.

### Proof

The part of the JNF connected with $\lambda =1$ is , by Fact 2.2. Since $\sigma (M) \subset \mathbb {R}_{+}$, we know from Fact 2.6 that a real logarithm of *M* exists. Let $M = \textrm{e}^R$ with real *R*, and consider the JNF of *R*. The only non-uniqueness emerges for the part that corresponds to  in the JNF of *M*, and this can be  or any real matrix that is (complex) similar to $\textrm{diag}(2 \pi \textrm{i}k, - 2 \pi \textrm{i}k)$ for some $0 \ne k \in \mathbb {Z}$; compare Remark 2.7.

None of the logarithms that emerge from $k\ne 0$ can lie in $\mathcal {A}^{(d)}_{0}$, since zero row sums for *R* means that 0 is an eigenvalue of *R*, then automatically with multiplicity 2, and *R* is real. So, only $k=0$ leads to a real logarithm of *M* from $\mathcal {A}^{(d)}_{0}$, which is the principal matrix logarithm, for instance in the form of a convergent series. It then has the same type of JNF, which implies that it has the same centraliser as *M* itself, and we get the uniqueness as claimed. $\square$

Let us close this section with a powerful consequence of Theorem 2.1, which was discussed and exploited in detail in Baake and Sumner (2022, Sec. 5, Thm. 5.3 and Cor. 5.5).

### Corollary 2.13

If $M\in \mathcal {M}_d$ is embeddable, so $M=\textrm{e}^Q$ for some generator *Q*, the latter commutes with *M*. When *M* is also cyclic, *Q* is an element of the non-unital real algebra generated by , with $\deg (q_{_A}) = d$. Thus, $Q \in \langle A, A^2, \ldots , A^{d-1}\rangle ^{}_{\mathbb {R}}$, which means

$$\begin{aligned} Q = \sum _{i=1}^{d-1} \alpha ^{}_{i} A^{i} \end{aligned}$$

for some $\alpha ^{}_{1}, \alpha ^{}_{2}, \ldots , \alpha ^{}_{d-1} \in \mathbb {R}$. In particular, if the spectral radius of *A* satisfies $\varrho _{_A} < 1$, such a representation exists for the convergent series . $\square$

Among the many results in the literature, the following existence and uniqueness result from Cuthbert (1972) sticks out because it is actually useful in practice.

### Theorem 2.14

Let $M\in \mathcal {M}_d$ satisfy the inequality $\,\min ^{}_{1\leqslant i \leqslant d} m^{}_{ii} > \frac{1}{2}$. Then,  with  is well defined as a converging series. Moreover, *M* is embeddable if and only if this *Q* is a generator, and no other embedding is possible.

### Proof

Under the assumption, the spectral radius of *A* satisfies $\varrho _{_A} < 1$, as a simple consequence of Gershgorin’s disk theorem (Horn and Johnson 2013, Thm. 6.1.1), see also Gantmacher (1986, Ch. 14.5). Then, there is some proper (that is, sub-multiplicative) matrix norm $\Vert .\Vert$ such that $\Vert A\Vert <1$, and the seriesis convergent in this norm, by a standard estimate in the form of a Weierstrass M-test. As all proper matrix norms on $\textrm{Mat}(d,\mathbb {C})$ are equivalent, convergence holds in any of them.

The limit clearly has zero row sums, but need not be a generator. When it is, we get $M=\textrm{e}^Q$ and *M* is embeddable, where the claimed uniqueness (and absence of any other candidate for a generator) follows from Cuthbert (1972, Cor. 1). $\square$

Another useful criterion for uniqueness is a consequence of the Corollary on p. 530 of Cuthbert (1973).

### Fact 2.15

Let $M\in \mathcal {M}_d$ be embeddable. If *M* also satisfies the condition

$$\begin{aligned} \det (M) \min _{1\leqslant i \leqslant d} m^{}_{ii} > \textrm{e}^{-\pi } \prod _{i=1}^{d} m^{}_{ii} , \end{aligned}$$

the embedding is unique. In particular, this is the case if $\det (M) > \textrm{e}^{-\pi } \approx 0.043{}214$. $\square$

This result is in line with several observations that a *logarithmic* scale would be more natural, which is sometimes referred to as ‘log det’ giving the proper intrinsic time scale of the problem (Goodman 1970). This can also be seen from the solution of the differential equation for the determinant of *M*(*t*); compare Eq. (34) below. In general, an embedding need not be unique. In such a case, one particular consequence of Theorem 2.1(5) is the following; see Davies (2010, Cor. 10) and Baake and Sumner (2020, Fact 2.14) for more.

### Corollary 2.16

If a cyclic matrix $M\in \mathcal {M}_d$ admits more than one embedding, the corresponding generators must commute with one another, and with *M*. $\square$

We are now ready to embark on the embedding problem for $d=3$ and $d=4$, where further notions will be introduced where and when we need them.

## Embedding in three dimensions

Given a general $M\in \mathcal {M}^{}_{3}$, where we know that 1 is an eigenvalue, it is most systematic to classify the cases according to $\deg (q_{_M})$, the degree of the minimal polynomial of *M*. This is an element of $\{ 1, 2, 3\}$, where $\deg (q_{_M}) = 1$ is trivial because this implies , which brings us back to Fact 2.9. From now on, it is often advantageous to work with , where $q_{_A} (x) = q_{_M} (x+1)$ implies $\deg (q_{_A}) = \deg (q_{_M})$. Recall that *A* (and hence *M*) is diagonalisable if and only if $q_{_A}$ has no repeated factor; see Horn and Johnson (2013, Cor. 3.3.8).

### Cases of degree 2

When $\deg (q_{_M}) = 2$, we must have $q_{_M} (x) = (x-1) (x-\lambda )$ with $\lambda \in [-1, 1)$, where embeddability excludes $\lambda = 0$ because $\det (M)$ must be positive; compare (Baake and Sumner 2020, Prop. 2.1). Since $q_{_M}$ has no repeated factor, the matrix *M* is always diagonalisable in this case. Now, we have to distinguish the two subcases $\mathfrak {m}_{\textsf{a}}(1) = 2$ and $\mathfrak {m}_{\textsf{a}}(\lambda ) = 2$.

If $\mathfrak {m}_{\textsf{a}}(1) = 2$, the JNF of *M* is $\textrm{diag}(1, 1, \lambda )$, with $\lambda \ne 1$. Since  is then similar to $\textrm{diag}(0,0,\lambda {-} 1)$, a quick calculation of $T \textrm{diag}(0,0,\lambda {-} 1) T^{-1}$ with $T\in \textrm{GL}(3, \mathbb {R})$ shows that *A* must be of the form

$$\begin{aligned} A = (\lambda - 1) \, ( \alpha ^{}_{1}, \alpha ^{}_{2}, \alpha ^{}_{3})^{\textsf{T}} \cdot (a^{}_{1}, a^{}_{2}, a^{}_{3}) \end{aligned}$$

subject to the condition $\sum _{i=1}^{3}\alpha ^{}_{i} a^{}_{i} = 1$ (to get the right eigenvalues), $\sum _{i=1}^{3} a^{}_{i} = 0$ (to have zero row sums), and the obvious sign conditions to make *A* a rate matrix. One thus finds that the most general Markov matrix here has the formwith the above conditions on the parameters, and subject to the remaining conditions that *M* is actually Markov. In particular, $|\alpha ^{}_{i} a^{}_{j} |\leqslant 1$ for all *i*, *j* together with $\alpha ^{}_{i} a^{}_{j}$ non-negative for $i=j$ and non-positive otherwise. Further calculation shows that this class comprises the matrices of the form $1 \oplus M'$ with $M'\in \mathcal {M}_2$ and its relatives that are obtained under a permutation of the three states, and all Markov matrices with a single non-trivial row.

Embeddability of *M* forces $\lambda \in (0,1)$ by the determinant condition, since we have $0 < \det (M) = \lambda$. Here, we are in the situation of Lemma 2.12, so we know that only one real logarithm of *M* exists, with JNF $\textrm{diag}\bigl (0,0,\log (\lambda )\bigr )$. Since  has spectral radius $\varrho _{_A} < 1$, we get7where the second identity is a consequence of $A^2 = (\lambda - 1) A$. Since $\lambda \in (0,1)$, the matrix *R* is always a generator.

Next, consider $$\mathfrak {m}_{\textsf{a}}(\lambda ) = 2$$ with $$- 1 \leqslant \lambda < 1$$ and $$\lambda \ne 0$$. Since  is similar to $$\textrm{diag}(0, \lambda {-}1, \lambda {-}1)$$, a general similarity transform shows that $$A\in \mathcal {A}^{(3)}_{0}$$ must be of the formagain with $$\sum _{i=1}^{3} a^{}_{i} = 0$$. Via a trivial re-parametrisation, one sees that the most general Markov matrices in this case are the *equal-input* matrices8where $$ C ( c^{}_{1}, c^{}_{2}, c^{}_{3} )$$ is the matrix with three equal rows $$( c^{}_{1}, c^{}_{2}, c^{}_{3} )$$ and summatory parameter $$c = c^{}_{1} + c^{}_{2} + c^{}_{3} = 1-\lambda $$, where $$c\ne 0$$ to exclude . Clearly, we need all $$c^{}_{i} \geqslant 0$$ together with $$c \leqslant 1 + c^{}_{i}$$ for all *i*; see Steel (2016) and Baake and Sumner (2020, 2022) for more on this class. Here, we know that uncountably many real logarithms exist, by Fact 2.6 and the discussion following it, and each of them must be diagonalisable by Fact 2.3. Let *R* be any of them.

When $\lambda > 0$, the SMT from Eq. (3) forces *R* to have eigenvalues 0 and $\mu ^{}_{\pm } = \log (\lambda ) \pm 2 \pi \textrm{i}k$ for some $k\in \mathbb {Z}$. When $k=0$, we have $\textrm{cent}(R) = \textrm{cent}(M)$, by the argument used in the proof of Lemma 2.12, which means that only one real logarithm corresponds to this choice, namely , which is a generator. Since $A^2 = (\lambda - 1) A$ holds also in this case, we get the formula from Eq. (7) again.

For each $k\ne 0$, the matrix *R* is simple, and $\textrm{cent}(R)$ is a true subalgebra of $\textrm{cent}(M)$. Consequently, the set of real logarithms corresponding to any such choice of *k* is uncountable: For every real logarithm *R*, also $SR S^{-1}$ is one, for arbitrary invertible $S \in \textrm{cent}(M)$. There can be further generators among them. So far, we have the following.

#### Lemma 3.1

Let $M\in \mathcal {M}^{}_{3}$ have $\deg (q_{_M} ) = 2$ and spectrum $\sigma (M) = \{ 1, \lambda \}$ with $\lambda \ne 1$. Then, $\lambda \in \mathbb {R}$, and we get the following case distinction.

1. If *M* has JNF $\textrm{diag}(1, 1, \lambda )$, it is embeddable if and only if $\lambda \in (0,1)$. In this case, one has the generator *Q* from Eq. (7), and the embedding $M=\textrm{e}^Q$ is unique.
2. If *M* has JNF $\textrm{diag}(1, \lambda , \lambda )$ with $\lambda >0$, hence $\lambda \in (0,1)$, it is embeddable with the generator *Q* from Eq. (7), but further solutions may exist. $\square$

Note that, in view of Eq. (8), the second claim of Lemma 3.1 is equivalent with the previous result from Baake and Sumner (2022, Prop. 2.12) that an equal-input Markov matrix with summatory parameter $0<c<1$ is always embeddable, even with an equal-input generator, where $\lambda = 1-c$. However, it need not be unique, as the following example shows.

#### Example 3.2

Let *M* be the equal-input matrix from Baake and Sumner (2020, Ex. 4.3), which has JNF $\textrm{diag}(1, \lambda , \lambda )$ with $\lambda = - \textrm{e}^{-\pi \sqrt{3}}<0$ and summatory parameter $c = 1+\textrm{e}^{-\pi \sqrt{3}} > 1$. Explicitly, it is  with $\delta = \frac{2}{3} \textrm{e}^{-\pi \sqrt{3}}$ and the matrix $J^{}_{3}$ from Lemma 6.13 below. This *M* is embeddable via two different circulant generators, as we shall derive later in Example 3.7.

Now, the matrix $M^2$ is still doubly embeddable in a circulant way, and is also still equal input, but now with summatory parameter $c' = c (2-c) = 1 - \textrm{e}^{-2 \pi \sqrt{3}} < 1$. Consequently, by Baake and Sumner (2020, Thm. 4.6), it is additionally embeddable via an equal-input generator,^1^ and we thus have an example with $c<1$ and three distinct embeddings. $\Diamond$

#### Remark 3.3

In the second case of Lemma 3.1, all other embeddings can be found with the help of Corollary 2.11, though none of them can be of equal-input type. Indeed, fixing a matrix $T\in \textrm{GL}(3,\mathbb {R})$ such that $M = T^{-1} \textrm{diag}(1,\lambda ,\lambda ) T$, one has to identify all generators among  with $k\in \mathbb {Z}$ and $x,y,z\in \mathbb {R}$ subject to $z>0$ and $yz-x^2=1$. In fact, by Casanellas et al. (2023, Lemma 3.1), one only has to consider integers *k* with

$$\begin{aligned} |k |\leqslant \frac{ |\log (\lambda ) |}{2 \pi \sqrt{3}} . \end{aligned}$$

In particular, if $\lambda > \textrm{e}^{- 2 \pi \sqrt{3}} \approx 1.877{}853 \cdot 10^{-5}$, there can be no further candidate except $k=0$, and the embedding via  is unique. This condition can also be expressed via the determinant, as $\det (M) = \lambda ^2 > \textrm{e}^{- 4 \pi \sqrt{3}} \approx 3.526{}333 \cdot 10^{-10}$, which is a further improvement over (Casanellas et al. 2023, Cor. 3.3 and Table 1) for this more special case. $\Diamond$

Finally, if *M* has a negative eigenvalue $\lambda$, this must have even multiplicity for embeddability, and *M* must be diagonalisable (due to Fact 2.6). So, it has $\deg (q_{_M})=2$, which is why this case occurs here. It was the last case for $d=3$ to be solved (Johansen 1974; Carette 1995). However, the answer is rather tricky and not practically useful, wherefore we do not recall those details. Instead, since the only Markov matrices with this type of JNF are the equal-input matrices from Eq. (8) with $c>1$, we can use their structure to get a simpler and constructive result. In fact, we also know $c\leqslant \frac{3}{2}$ in this case, since $c\leqslant 1 + c^{}_i$ for all *i*, hence $-\frac{1}{2}\leqslant \lambda < 0$. The key now is to control the matrices that commute with *M*.

If an equal-input matrix $M_c$ has a real logarithm, so $M_c = \textrm{e}^R$ for some matrix $R\in \textrm{Mat}(3,\mathbb {R})$, one has . If  with $C=C(c^{}_{1}, c^{}_{2}, c^{}_{3})$ and $c=c^{}_{1} + c^{}_{2} + c^{}_{3}$ as above, one clearly has  if and only if . Since we are only interested in matrices *R* with zero row sums, we define the *commutant* of $M_c$ aswhere one has $\textrm{comm}(M_c) = \textrm{comm}(C)$ with the *C* from above. Clearly, one has the trivial case that , which is a six-dimensional algebra, while it is four-dimensional for all other equal-input matrices, as can be derived from their diagonal form. In view of the situation at hand, we now look more closely at the case that all $c^{}_{i} > 0$.

#### Lemma 3.4

Let $M_c$ be the matrix from Eq. (8), with all $c^{}_i > 0$. Then, the algebra $\textrm{comm}(M_c)$ is generated by the four matrices

$$\begin{aligned} \begin{pmatrix} 0 &{} 0 &{} 0 \\ 0 &{} * &{} c^{}_{3} \\ 0 &{} c^{}_{2} &{} * \end{pmatrix}, \; \begin{pmatrix} * &{} 0 &{} c^{}_{3} \\ 0 &{} 0 &{} 0 \\ c^{}_{1} &{} 0 &{} * \end{pmatrix}, \; \begin{pmatrix} * &{} c^{}_{2} &{} 0 \\ c^{}_{1} &{} * &{} 0 \\ 0 &{} 0 &{} 0 \end{pmatrix} \quad \text {and}\quad \begin{pmatrix} * &{} \alpha &{} -\gamma \\ -\alpha &{} * &{} \beta \\ \gamma &{} -\beta &{} * \end{pmatrix}, \end{aligned}$$

with $\alpha = (c^{}_{1} + c^{}_{3})(c^{}_{2} + c^{}_{3})$, $\beta =(c^{}_{1} + c^{}_{2})(c^{}_{1} + c^{}_{3})$ and $\gamma =(c^{}_{1} + c^{}_{2})(c^{}_{2} + c^{}_{3})$. Here, $*$ always denotes the unique real number to assure row sum 0. These matrices are linearly independent over $\mathbb {R}$, and $\textrm{comm}(M_c)$ is a subalgebra of $\mathcal {A}^{(3)}_{0}$ of dimension 4.

#### Proof

Let $C=C(c^{}_{1}, c^{}_{2}, c^{}_{3})$ and observe that  holds for every *R* with zero row sums. Then,  means , which holds if and only if $(c^{}_{1}, c^{}_{2}, c^{}_{3})$ is a left eigenvector of *R* with eigenvalue 0.

This eigenvector property is satisfied for each of the four matrices given, as one can check by a simple calculation. As $c^{}_{i} > 0$ for all *i*, the four matrices are all non-zero and indeed linearly independent over $\mathbb {R}$, wherefore they span a four-dimensional subalgebra of $\mathcal {A}^{(3)}_{0}$, which must be contained in $\textrm{comm}(M_c)$.

Since $\mathcal {A}^{(3)}_{0}$ has dimension 6, and since the left eigenvector condition results in two independent constraints due to the zero row sum property of *R*, we see that $\textrm{comm}(M_c)$ has dimension 4, and is thus spanned by the matrices given. $\square$

The commutant can also be calculated explicitly for any non-zero vector $(c^{}_{1}, c^{}_{2}, c^{}_{3})$ via the left eigenvector condition, and always gives a four-dimensional commutant, then with a slightly more complicated parametrisation; we skip further details at this point.

#### Lemma 3.5

Let $M_c$ be the matrix from Eq. (8), with all $c^{}_i \geqslant 0$ and $c>0$, and assume that $Q \in \textrm{comm}(M_c)$ is a generator such that $\textrm{e}^Q$ is an equal-input matrix. Then, we have  for some $r\geqslant 0$, with the matrix *C* and summatory parameter *c* from $M_c$.

#### Proof

If $\textrm{e}^Q$ is of equal-input type, we have  with $\widetilde{C} = C (r^{}_{1}, r^{}_{2}, r^{}_{3})$ and all $r_i \geqslant 0$, where *r* is the summatory parameter. Since *Q* commutes with $M_c$ by assumption, we obtain , as we explained prior to Fact 2.3, and thus . As $C \widetilde{C} = c \widetilde{C}$ and $\widetilde{C} C = r C$, we get $\widetilde{C} = \frac{r}{c} C$ and the claim follows. $\square$

Our strategy is now the following. Since we only need further insight into the embedding properties of $M_c$ with $1 < c \leqslant \frac{3}{2}$, we assume $c>1$, which forces $c_i > 0$ for all *i*. So, consider the matrix $C = C(c^{}_{1}, c^{}_{2}, c^{}_{3})$, and let $R \in \textrm{comm}(C)$ be a matrix such that $\textrm{e}^R$ is equal input, hence , where we know from Lemma 3.5 that $\widetilde{C} = \frac{r}{c} C$ for some $0 \leqslant r \ne 1$, which is the new summatory parameter. Since the cases with $r\in [0,1)$ are trivially embeddable, we now concentrate on the case $r>1$ and determine the maximal value for embeddability, $r^{\max }$. This way, we will find the full embeddability range $[0,1) \cup (1, r^{\max } ]$ for the direction defined by *C*, respectively by $(c^{}_{1}, c^{}_{2}, c^{}_{3})$.

Given $(c^{}_{1}, c^{}_{2}, c^{}_{3})$ with $c>1$ as above, let $Q^{}_1, Q^{}_2, Q^{}_3$ and $R^{}_{0}$ denote the four matrices from Lemma 3.5, in the order as given there. All $Q^{}_i$ are generators, while $R^{}_{0} \in \mathcal {A}^{(3)}_{0}$ never is. It is immediate that $Q=xQ^{}_1 + yQ^{}_2 + zQ^{}_3 + w R^{}_0$ is a generator if and only if9In particular, $x,y,z\geqslant 0$, and they are even strictly positive unless $w=0$.

#### Lemma 3.6

Consider $Q = xQ^{}_1 + yQ^{}_2 + zQ^{}_3 + w R^{}_{0}$ with the matrices from Lemma 3.4 as defined above. Assume that *Q* is a generator so that $M=\textrm{e}^Q$ has a double negative eigenvalue, say $-\lambda < 0$. Then, *M* is an equal-input matrix with parameters $\frac{r}{c} (c^{}_{1}, c^{}_{2}, c^{}_{3})$ for some $r>1$.

#### Proof

Since $\sigma (M) = \{ 1, -\lambda , -\lambda \}$ by assumption, this time viewed as a multi-set, and since *Q* is a real matrix, we must have $\sigma (Q) = \{ 0, \eta \pm (2 k{+}1) \pi \textrm{i}\}$ for some $k \in \mathbb {N}_0$ by the SMT from Eq. (3), where $\eta <0$. So, *Q* is simple and hence diagonalisable, with $\lambda = \textrm{e}^{\eta }$. Clearly, *M* is then diagonalisable as well, and its JNF is $\textrm{diag}(1, -\lambda , -\lambda )$.

Now, we can repeat the little calculation that led to Eq. (8) to conclude that *M* is indeed equal input. Since we know that *Q* commutes with the original *C*-matrix defined by the specified $c_i$, an application of Lemma 3.5 establishes the claim. $\square$

This means that we can proceed via the SMT. If *Q* is the generator from Lemma 3.6, its spectrum is $\sigma (Q) = \{ 0, -\Delta \pm s \}$ with

10 $$\begin{aligned} \Delta&= \frac{1}{2} \bigl ( (c^{}_{2} + c^{}_{3} ) x + (c^{}_{1} + c^{}_{3}) y + (c^{}_{1} + c^{}_{2} ) z \bigr ) \geqslant 0 \quad \text {and} \nonumber \\ s^2&= \frac{1}{4} \bigl ( (c^{}_{1} + c^{}_{3}) (x-y) - (c^{}_{1} + c^{}_{2}) (x-z) \bigr )^2 + c^{}_{2} c^{}_{3} (x-y)(x-z) \nonumber \\&\ \quad + (c^{}_{1} + c^{}_{2} + c^{}_{3}) \bigl ( c_1^2 (z-y) + c_2^2 (x-z) + c_3^2 (y-x) \bigr ) w\nonumber \\&\ \quad - 2 (c^{}_{1} + c^{}_{2} + c^{}_{3}) (c^{}_{1} + c^{}_{2})(c^{}_{1} + c^{}_{3}) (c^{}_{2} + c^{}_{3}) w^2 \nonumber \\&=:\chi + \psi w + \varphi w^2, \end{aligned}$$

as can easily be checked with any computer algebra system. Note that $s^2$ only depends on differences of the parameters *x*, *y*, *z*, which will become important shortly. Now, we fix $s^2 = - (2 k+1)^2 \pi ^2$ for some $k\in \mathbb {N}_0$, which gives $\textrm{e}^{\pm s} = -1$, and $\sigma (\textrm{e}^Q)$ thus is the multi-set $\{ 1, -\lambda , -\lambda \}$ with $\lambda = \textrm{e}^{-\Delta }$. We need the largest possible value of $\lambda$ and thus the smallest value for $\Delta$, where all inequalities from (9) have to be satisfied for *Q* to be a generator. When $w=0$, we see that $s^2=\chi$ is the sum of a square and one extra term, which can be done in three different ways (only one of which is shown above). The other two choices give $(y-x)(y-z)$ and $(z-x)(z-y)$ in the extra term, respectively, which ensures positivity according to $\max (x,y,z)$. This implies $s^2 \geqslant 0$ whenever $w=0$. So, we need $w\ne 0$ and then $x,y,z > 0$ to ensure that *Q* is a generator.

As $c^{}_{i} >0$ for all *i*, no partial derivative of $\Delta$ can vanish (except the trivial one with respect to *w*), while those of $s^2$ are identically 0, because $s^2 = - (2k+1)^2 \pi ^2$ is constant. The minimum we are looking for, as a function of *x*, *y*, *z*, is then not a local one, but must lie on the boundary of the region that guarantees the generator property of *Q*. We thus need the correct value for *w*. Now, $s^2 = - (2k+1)^2 \pi ^2$ leads to a quadratic equation for *w*, with the two solutions

$$\begin{aligned} w = \frac{-1}{2 \varphi } \Bigl (\psi \pm \sqrt{\psi ^2 - 4 \varphi \bigl ( \chi + (2k+1)^2 \pi ^2 \bigr )}\, \Bigr ), \end{aligned}$$

where $\varphi , \psi , \chi$ are defined in (10).

#### Example 3.7

Consider the special case that $c^{}_{1} = c^{}_{2} = c^{}_{3} = \frac{c}{3} > 0$, where $\alpha = \beta = \gamma = \frac{4}{9} c^2$ and then $\varphi = - \frac{16}{27} c^4$, $\psi = 0$ and $\chi = \frac{c^2}{9} \bigl ( (x-y)^2 + (x-z)^2 - (x-y)(x-z)\bigr )$. Now, the condition $w_x = w_y = w_z = 0$ for a stationary point is equivalent with $x=y=z$, which gives $\chi = 0$ and thus the two solutions

$$\begin{aligned} w = \pm \frac{9 (2k{+}1) \pi }{4 c^2 \sqrt{3}} , \end{aligned}$$

where we now choose $k=0$ to minimise the modulus of *w*. Since $x\geqslant \frac{ \pi \sqrt{3} }{ c }$ from (9), the minimal value of $\Delta$ is $\Delta ^{\min } = c x = \pi \sqrt{3}$, which means $\lambda = - \textrm{e}^{-\Delta ^{\min }} = 1 - r^{\max }$ and hence $r^{\max } = 1 + \textrm{e}^{-\pi \sqrt{3}}$, which we know from Example 3.2.

There are two solutions for the generator *Q* now, namely the circulant matrices

$$\begin{aligned} \frac{2\pi }{\sqrt{3}}\begin{pmatrix} -1 &{} 1 &{} 0 \\ 0 &{} -1 &{} 1 \\ 1 &{} 0 &{} -1 \end{pmatrix} \quad \text {and} \quad \frac{2\pi }{\sqrt{3}}\begin{pmatrix} -1 &{} 0 &{} 1 \\ 1 &{} -1 &{} 0 \\ 0 &{} 1 &{} -1 \end{pmatrix}. \end{aligned}$$

One indeed gets  with $\delta = \frac{2}{3} \textrm{e}^{-\pi \sqrt{3}}$ and $J^{}_{3}$ as in Lemma 6.13 below. This extremal case is known from Baake and Sumner (2020, Ex. 4.3), see also Example 3.2, but here we directly obtain *two* embeddings. $\Diamond$

Let us now sketch the general argument. Select a parameter triple $(c^{}_1, c^{}_2, c^{}_3 )$ in the positive cone, subject to the condition $c>1$. We now determine the maximal point on the ray defined by this triple that is still embeddable. To this end, we fix $s^2 = - (2k+1)^2\pi ^2$ with $k\in \mathbb {N}_0$, so $M_c$ has two negative eigenvalues. This gives an implicit equation for *w* as a function of *x*, *y*, *z*. The stationarity condition $w_x = w_y = w_z = 0$, which we will justify a little later, now gives that both $x-y$ and $x-z$ are proportional to *w*, the details of which are best calculated with a computer algebra system. Inserting this into $s^2$ leads to

$$\begin{aligned} s^2 = - \frac{c (c^{}_1 + c^{}_2)^2 (c^{}_1 + c^{}_3)^2 (c^{}_2 + c^{}_3)^2}{4 c^{}_1 c^{}_2 c^{}_3} w^2 = - (2k+1)^2 \pi ^2, \end{aligned}$$

and hence to the always real solutions (with $k\in \mathbb {N}_0$)

11 $$\begin{aligned} w = \pm \frac{2 (2 k{+}1) \pi \, \sqrt{ \frac{c^{}_{1_{}} c^{}_2 c^{}_3}{c} } }{(c^{}_1 + c^{}_2) (c^{}_1 + c^{}_3) (c^{}_2 + c^{}_3)} . \end{aligned}$$

This can now again be inserted into the expressions for $x-y$ and $x-z$ to eliminate *w*, in two different ways, for the two choices of the sign of *w*.

Next, to minimise $\Delta$, we want to take the *minimal* value of *x*, *y*, and *z* that is allowable by the generator condition (9). This forces $k=0$, but which of the three conditions in (9) is pivotal here depends on the parameters $c_i$. For *x*, the condition reads

$$\begin{aligned} x \geqslant (c^{}_1 + c^{}_2) (c^{}_1 + c^{}_3) \max \left( \frac{w}{c^{}_{2}}, \frac{-w}{c^{}_{3}} \right) . \end{aligned}$$

Taking the minimal value and determining *y* and *z* from the stationarity condition then gives $\Delta \geqslant \pi \sqrt{c} \, c^{}_{1} / \! \sqrt{c^{}_{1} c^{}_2 c^{}_3}$ upon inserting, and similarly for the other two paths that start from *y* or from *z*. Since all conditions have to be satisfied to obtain a generator, we find

12 $$\begin{aligned} \Delta ^{\min } = \frac{\pi \kappa \sqrt{c}}{\sqrt{c^{}_{1} c^{}_2 c^{}_3}}, \quad \text {with}\quad \kappa = \max (c^{}_1, c^{}_2, c^{}_3) , \end{aligned}$$

which holds for *both* choices $w = \pm |w |$. The corresponding two generators read

13 $$\begin{aligned} Q^{}_{\pm } = \frac{\pi }{\sqrt{(c^{}_1 + c^{}_2 + c^{}_3) c^{}_1 c^{}_2 c^{}_3 }\,} \begin{pmatrix} -\kappa (c^{}_2 + c^{}_3) &{} c^{}_2 (\kappa \pm c^{}_3) &{} c^{}_3 (\kappa \mp c^{}_2) \\ c^{}_1 (\kappa \mp c^{}_3) &{} -\kappa (c^{}_1 + c^{}_3) &{} c^{}_3 (\kappa \pm c^{}_1) \\ c^{}_1 (\kappa \pm c^{}_2) &{} c^{}_2 (\kappa \mp c^{}_1) &{} -\kappa (c^{}_1 + c^{}_2) \end{pmatrix}, \end{aligned}$$

which is again best checked by a computer algebra system. For $c^{}_1 = c^{}_2 = c^{}_3 = \frac{c}{3}$, this reduces to the matrices from Example 3.7 for the constant-input case. Note that the expressions for $\Delta ^{\min }$ and for $Q^{}_{\pm }$ are positively homogeneous of degree 0 in the $c^{}_{i}$, so only depend on the *direction* defined by the vector $(c^{}_1, c^{}_2, c^{}_3 )$.

The final step now consists in observing that the relation $M_c = \exp (Q^{}_{\pm })$, via the negative eigenvalue $\lambda = - \textrm{e}^{-\Delta ^{\min }}$ and $c = 1 - \lambda$, gives the consistency condition $c = 1+ \textrm{e}^{-\Delta ^{\min }}$. If this is not satisfied, one has to replace the $c^{}_i$ by the unique values

$$\begin{aligned} c^{\max }_{i} = \frac{1 + \textrm{e}^{-\Delta ^{\min }}}{c^{}_1 + c^{}_2 + c^{}_3} \, c^{}_{i} , \end{aligned}$$

which give the extremal point of embeddability on the ray defined by $(c^{}_1, c^{}_2, c^{}_3 )$. We thus have the following constructive counterpart to the results from Johansen (1974) and Carette (1995).

#### Proposition 3.8

An equal input matrix $M_c \in \mathcal {M}^{}_3$ with parameter triple $(c^{}_1, c^{}_2, c^{}_3 )$ subject to $c^{}_{i} > 0$ and $c > 1$ is embeddable if and only if $c \leqslant 1 + \textrm{e}^{-\Delta ^{\min }}$ holds with $\Delta ^{\min }$ from Eq. (12). In the extremal case, one has $M_c = \exp (Q^{}_{\pm })$ with the two generators from Eq. (13). Further, one has $\Delta ^{\min } \geqslant \pi \sqrt{3}$ and thus $c^{\max }\leqslant 1+\textrm{e}^{-\pi \sqrt{3}}$.

#### Proof

For the maximal point on the ray, the claim follows from our above constructive calculation, once we show that this really corresponds to a minimum (if $w>0$) or a maximum (if $w<0$). The Hessian can be calculated, and reads

$$\begin{aligned} H = \frac{\pm \sqrt{\frac{c^{}_{1_{}} c^{}_2 c^{}_3}{c^{}_1 + c^{}_2 + c^{}_3}}}{2 \pi (c^{}_1 {+} c^{}_2)(c^{}_1 {+} c^{}_3) (c^{}_2 {+} c^{}_3)} \begin{pmatrix} (c^{}_2 + c^{}_3)^2 &{} c^{}_1 c^{}_2 - c c^{}_3 &{} c^{}_1 c^{}_3 - c c^{}_2 \\ c^{}_1 c^{}_2 - c c^{}_3 &{} (c^{}_1 + c^{}_3 )^2 &{} c^{}_2 c^{}_3 - c c^{}_1 \\ c^{}_1 c^{}_3 - c c^{}_2 &{} c^{}_2 c^{}_3 - c c^{}_1 &{} (c^{}_1 + c^{}_2)^2 \end{pmatrix} \end{aligned}$$

for the two signs of *w*. It is positive (resp. negative) semi-definite, with one zero eigenvalue and two positive (resp. negative) ones. The neutral direction is $(1,1,1)^{\textsf{T}}$, so we have a valley of minima (maxima) along this direction, relative to the two remaining degrees of freedom, as *H* does not change along the valley. Both signs of *w* give the same value for $\Delta ^{\min }$, as claimed.

It remains to see that every point on our ray with $1< c < c^{\max } = 1 + \textrm{e}^{-\Delta ^{\min }}$ is also embeddable. But this is now evident, because we can replace the above (optimal) values for *x*, *y*, *z* by $x+\theta , y+\theta , z+\theta$ with any $\theta >0$ without compromising the generator condition. Since $\Delta$ is linear in *x*, *y*, *z*, we can thus obtain any value of $\Delta$ in the interval $[\Delta ^{\min }, \infty )$, and the claimed embeddability follows.

Finally, consider $\Delta ^{\min }$ from Eq. (12), with $c^{}_{1} \geqslant c^{}_{2} \geqslant c^{}_{3} >0$. It is a simple exercise to check that it reaches its minimum whenever the three $c_i$ are equal, which then is the value we saw in Example 3.7, with the corresponding value for $c^{\max }$ as stated. $\square$

#### Remark 3.9

By construction, the generators $Q^{}_{\pm }$ from Eq. (13) commute with the matrix $C = C(c^{}_{1}, c^{}_{2}, c^{}_{3})$ for the fixed parameter triple, so also  for the rate matrix . Consequently, for $\tau \geqslant 0$, we obtain

$$\begin{aligned} \exp (Q^{}_{\pm } + \tau Q^{}_{C}) = \exp (\tau Q^{}_{C}) \exp (Q^{}_{\pm }) = \exp (\tau Q^{}_{C}) M_{c^{\max }} , \end{aligned}$$

which is an equal-input matrix with summatory parameter

$$\begin{aligned} c(\tau ) = 1 + \textrm{e}^{-\tau c^{\max }} (c^{\max }-1) . \end{aligned}$$

With $\tau \geqslant 0$, we thus reach all values in $(1, c^{\max }]$, and always get two embeddings.

Now, this was based on the choice $k=0$, and one can now repeat the exercise for any $k\in \mathbb {N}$ in Eq. (11). For smaller and smaller values of $c>1$, one thus obtains further embeddings, where two new embeddings occur at

$$\begin{aligned} \Delta = \frac{(2k+1) \pi \kappa \sqrt{c}}{\sqrt{c^{}_{1} c^{}_2 c^{}_3}} \end{aligned}$$

for each $k\in \mathbb {N}$. We leave further details to the interested reader. $\Diamond$

Let us now turn our attention to the generic situation for $d=3$.

### Cyclic cases

When $\deg (q_{_M}) = 3$, we know that *M* is cyclic. If *M* is embeddable, no eigenvalue can lie on the (closed) negative real axis, by an application of Culver’s criterion (Fact 2.6). Here, the spectrum of *M* can be real or not, which we now consider separately.

When $\sigma (M) \subset \mathbb {R}$, we are in the situation of Theorem 2.5, so all eigenvalues must be positive for potential embeddability. Then, the spectral radius of  satisfies $\varrho _{_A} < 1$, and it remains to formulate an effective condition for when the well-defined matrix  from (5) is a generator. In this situation, by Theorem 2.1 and Corollary 2.13, we know that  with $\alpha , \beta \in \mathbb {R}$. Now, there are still two cases.

When *A* is diagonalisable, we have $\sigma (A) = \{ 0, \mu , \nu \}$ with $\mu , \nu \in (-1,0)$ and $\mu \ne \nu$. Then, it follows from the SMT (see (3) and Baake and Sumner 2022, Eq. (4.6) for details) that

14 $$\begin{aligned} \alpha = \frac{\mu ^2 \log (1+\nu ) - \nu ^2 \log (1+\mu )}{\mu \nu (\mu -\nu )} \quad \text {and} \quad \beta = \frac{-\mu \log (1+\nu ) + \nu \log (1+\mu )}{\mu \nu (\mu -\nu )} , \end{aligned}$$

which is the unique solution to the SMT-induced equation

$$\begin{aligned} \begin{pmatrix} \mu &{} \mu ^2 \\ \nu &{} \nu ^2 \end{pmatrix} \begin{pmatrix} \alpha \\ \beta \end{pmatrix} \, = \, \begin{pmatrix} \log (1+\mu ) \\ \log (1+\nu ) \end{pmatrix}. \end{aligned}$$

Otherwise, when *A* is cyclic but fails to be diagonalisable, its JNF must be $0 \oplus \mathbb {J}^{}_{2} (\mu )$ with $\mu \in (-1,0)$, and Baake and Sumner (2022, Eq. (4.7)) then gives

15 $$\begin{aligned} \alpha =\frac{2\log (1+\mu )}{\mu } - \frac{1}{1+\mu } \quad \text {and} \quad \beta = \frac{1}{\mu (1 + \mu )} - \frac{\log (1+\mu )}{\mu ^2} , \end{aligned}$$

which also emerges from (14) as a limit of de l’Hospital type. This gives the following result.

#### Proposition 3.10

Let $M\in \mathcal {M}^{}_{3}$ by cyclic, with real spectrum. Then, *M* is embeddable if and only if the following two conditions are satisfied, where  as before.

1. All eigenvalues of *M* are positive, which automatically implies $\varrho _{_A} <1$.
2. The real matrix  is a generator, where $Q=\alpha A + \beta A^2$ with $\alpha , \beta$ from Eqs. (14) or (15), depending on whether *M* is diagonalisable or not.

In this case, the embedding is unique. $\square$

Otherwise, we have $\sigma (M) = \{ 1, \lambda , \overline{\lambda } \}$ with $\lambda \in \mathbb {C}{\setminus } \mathbb {R}$, but we can still use Corollary 2.13 for the generator *Q*. Here,  with , which implies $\textrm{tr}(Q) <0$ and thus $|\lambda |^2 = \det (M) = \textrm{e}^{\textrm{tr}(Q)} < 1$. Then, $z^{}_{0} :=\log (\lambda )$ via the standard branch of the complex logarithm is well defined, and $z^{}_{k} = z^{}_{0} + k \, 2 \pi \textrm{i}$ with $k\in \mathbb {Z}$ runs through *all* complex logarithms of $\lambda$. Now, setting $\lambda = 1+\mu$ and writing $Q=\alpha A + \beta A^2$, the SMT leads to the conditions

$$\begin{aligned} \begin{pmatrix} \exp \bigl (\alpha \mu + \beta \mu ^2 \bigr ) \\ \exp \bigl ( \alpha \overline{\mu } + \beta {\overline{\mu } }^2 \bigr )\end{pmatrix} = \begin{pmatrix} \lambda \\ \overline{\lambda } \end{pmatrix}, \end{aligned}$$

for which we need a *real* solution $\alpha ,\beta$. Upon taking logarithms, one can quickly check that this is only possible if we use a complex-conjugate pair of them for the two lines, hence

$$\begin{aligned} \begin{pmatrix} \mu \;\; \mu ^2 \\ \overline{\mu } \;\; \overline{\mu } ^2 \end{pmatrix} \begin{pmatrix} \alpha \\ \beta \end{pmatrix} = \begin{pmatrix} z^{}_{k} \\ \overline{z^{}_{k} } \end{pmatrix} \end{aligned}$$

for some $k\in \mathbb {Z}$. This is uniquely solved by

16 $$\begin{aligned} \begin{pmatrix} \alpha ^{}_{k} \\ \beta ^{}_{k} \end{pmatrix} = \frac{1}{|\mu |^2 (\overline{\mu } - \mu )} \, \begin{pmatrix} \overline{\mu } ^2 z^{}_{k} - \mu ^2 \overline{z^{}_{k} } \\ -\overline{\mu } z^{}_{k} + \mu \overline{z^{}_{k} } \end{pmatrix}, \end{aligned}$$

which is indeed real. So, for each choice of $k\in \mathbb {Z}$, our matrix *M* has precisely *one* real logarithm, namely $\alpha ^{}_{k} A + \beta ^{}_{k} A^2 \in \textrm{Mat}(3, \mathbb {R})$. We can summarise this as follows.

**Table 1:** Summary of embedding cases for $d=3$, where we use MG as abbreviation for the property to be a Markov generator

$\deg (q_{_M})$	JNF	condition(s)	unique	details
1		–	Yes	Fact 2.9
2	$$ \begin{array}{l}\textrm{diag}(1,1,\lambda ) \\ \lambda \ne 1 \end{array}$$	$\lambda \in (0,1)$	Yes	Lemma 3.1(1)
	$$\begin{array}{l} \textrm{diag}(1,\lambda ,\lambda ) \\ \lambda > 0 \end{array}$$	$\lambda \in (0,1)$	$$\begin{array}{l} \text {Not} \\ \text {Always} \end{array}$$	Lemma 3.1(2)
	$$\begin{array}{l} \textrm{diag}(1,\lambda ,\lambda ) \\ \lambda < 0 \end{array}$$	$\lambda \in [-\textrm{e}^{-\pi \sqrt{3}}, 0)$	No	Prop. 3.8
3	$$\begin{array}{l} \textrm{diag}(1,\lambda _1,\lambda _2) \\ 1 \ne \lambda _1 \ne \lambda _2 \ne 1 \end{array}$$		Yes	Prop. 3.10
	$$\begin{array}{l} 1 \oplus \mathbb {J}_2 (\lambda ) \\ \lambda \ne 1 \end{array}$$		Yes	Prop. 3.10
	$$\begin{array}{l} \textrm{diag}(1, \lambda , \overline{\lambda }\, )\\ \lambda \in \mathbb {C}{\setminus } \mathbb {R}\end{array}$$	$$\begin{array}{l} |\lambda |\in (0,1) \\ R_k is MG for some k \end{array}$$	$$\begin{array}{l} \text {Not} \\ \text {Always} \end{array}$$	Prop. 3.11

#### Proposition 3.11

Let $M\in \mathcal {M}^{}_{3}$ have spectrum $\sigma (M) = \{1, \lambda , \overline{\lambda } \}$ with $\lambda \in \mathbb {C}{{\setminus }} \mathbb {R}$. Then, *M* is embeddable if and only if the following two conditions are satisfied, with .

1. One has $$0< |\lambda |< 1$$.
2. There is a $k\in \mathbb {Z}$ such that the real logarithm $R^{}_{k} =\alpha ^{}_{k} A + \beta ^{}_{k} A^2$ of $M$, with $\alpha ^{}_{k}$ and $\beta ^{}_{k}$ from Eq. (16), is a generator, which then gives $M=\textrm{e}^{Q}$ with $Q=R^{}_{k}$.

In this case, $R^{}_{k}$ is a generator for only finitely many $k\in \mathbb {Z}$, and no other candidates exist. The number of solutions is bounded by the integer $\big \lfloor 1 - \frac{\log (\det (M))}{2 \pi \sqrt{3}}\big \rfloor$.

#### Proof

The first condition was derived in the paragraph after Proposition 3.10.

The second condition follows from our above calculation, which also constructs all possible candidates for the real logarithms of *M*. That only finitely many of them can be generators follows once again from the bounds on the imaginary parts of their eigenvalues, as is detailed in Casanellas et al. (2023, Thm. 4.5), including the bound as stated. $\square$

Multiple embeddings do occur, though not for sufficiently large values of $\det (M)$. Also, Theorem 2.14 can often be used to exclude non-uniqueness. Explicit examples with multiple generators are discussed in Speakman (1967), which considers an equal-input example in our terminology, and in Davies (2010, Ex. 17), which fits Proposition 3.11; see also Baake and Sumner (2020, Rem. 5.5), and Cuthbert (1973) for a more general discussion of multiple embeddings. The basic results of this section on $d=3$ are summarised in Table 1.

## Embedding in four dimensions

If $M \in \mathcal {M}_4$ has $\deg (q_{_M}) = 1$, we are back to  and Fact 2.9, hence to . So, we need to consider the cases $\deg (q_{_M}) \in \{ 2,3,4\}$.

### Cases of degree 2

In view of Fact 2.2, there are three possible cases here, all of which are diagonalisable, again because their minimal polynomial has no repeated factor. First, let us look at $M\in \mathcal {M}_4$ with JNF $\textrm{diag}(1, 1, 1, \lambda )$ and $\lambda \ne 1$, where $0 < \det (M) \leqslant 1$ then forces $\lambda \in (0,1)$. This class of matrices is erroneously claimed to be impossible in Casanellas et al. (2023, Lemma 5.1), as can be seen from looking at17with $a,b \in [0,1]$. Due to the block structure, one can invoke Theorem 2.4 to see that *M* is embeddable if and only if $0 \leqslant a + b < 1$. More generally, in complete analogy to the corresponding case in three dimensions from Sect. 3.1, the most general form of *M* issubject to the conditions $\sum _{i=1}^{4} \alpha ^{}_{i} a^{}_{i} = 1$ and $\sum _{i=1}^{4} a^{}_{i} = 0$, and to the restrictions in sign and absolute value to guarantee that *M* is Markov. Beyond the matrix in (17), and its obvious relatives that emerge from simultaneous permutations of rows and columns, one can also get

18 $$\begin{aligned} M = \begin{pmatrix} \lambda &{} x &{} y &{} z \\ 0 &{} 1 &{} 0 &{} 0 \\ 0 &{} 0 &{} 1 &{} 0 \\ 0 &{} 0 &{} 0 &{} 1 \end{pmatrix} \end{aligned}$$

with $\lambda =1-x-y-z$ and $x,y,z\geqslant 0$ subject to the condition that $0< x+y+z < 1$, so that *M* is invertible and $\lambda \in (0,1)$. As before, all other cases of this kind are obtained by simultaneous permutations of rows and columns. To see that these are all possibilities (with $\lambda \ne 1$), one uses the constraints on the $a_i$ and $\alpha _j$ to show that at most one $\alpha _i < 0$ and at most one $\alpha _j > 0$. Then, a simple case distinction, which we leave to the interested reader, gives the above characterisation of this class.

In any case of this class, with $\lambda \in (0,1)$,  is similar to $\textrm{diag}(0,0,0,\lambda {-}1)$, hence $A^2 = (\lambda - 1) A$. This means that  is once again given by Eq. (7). Since this is a generator, *M* is embeddable. As $M=\textrm{e}^R$ implies that *R* is diagonalisable, there are also real logarithms with spectrum $\sigma (R) = \{ 0, \pm 2\pi \textrm{i}k, \log (\lambda ) \}$ for $k\in \mathbb {N}$. Among them, there can be further generators (but at most finitely many), and the embedding need not be unique.

An analogous situation is met for *M* with JNF $\textrm{diag}(1, \lambda , \lambda , \lambda )$ and $\lambda \ne 1$. As in the three-dimensional case before Eq. (8), via a completely analogous calculation, one can check that the most general Markov matrices of this type are the equal-input matriceswith parameters $c^{}_{i} \geqslant 0$ and $c = 1-\lambda = c^{}_{1} + \cdots + c^{}_{4} > 0$; see also Casanellas et al. (2023).

Here, we must have $\lambda \in (0,1)$ for embeddability, again due to the determinant condition $0 < \det (M) \leqslant 1$ (and also already by Fact 2.6), hence $c=1-\lambda \in (0,1)$ as well. Once again, $A^2 = (\lambda - 1)A$ and $\log (1+A)$ from (7) is a generator, so $M_c$ is embeddable; compare also with Casanellas et al. (2023, Prop. 5.17). Other real logarithms of $M_c$ exist, then with $\sigma (R) = \{ 0, \log (\lambda ), \log (\lambda ) \pm 2\pi \textrm{i}k \}$ for $k\in \mathbb {N}$, some of which might be generators. So far, we have the following.

#### Lemma 4.1

Let the JNF of $M\in \mathcal {M}_4$ be $\textrm{diag}(1, 1, 1, \lambda )$ or $\textrm{diag}(1, \lambda , \lambda , \lambda )$, with $\lambda < 1$. Then, we have $\deg (q_{_M}) = 2$, and the following conditions are equivalent.

1. The matrix *M* is embeddable.
2. One has $\det (M) > 0$, which is equivalent to $\lambda \in (0,1)$.
3. The matrix  is a generator.

In this case, we get $Q = \frac{-\log (\lambda )}{1-\lambda } A$, but the embedding need not be unique. $\square$

Let us note that there is a connection of the second case with Remark 4.6 via taking square roots, which is another instance of the subtle complications that may show up.

It remains to consider the JNF $\textrm{diag}(1, 1, \lambda , \lambda )$, where $\det (M)>0$ no longer implies $\lambda$ to be positive. If $\lambda >0$, we get embeddability with $Q = - \frac{\log (\lambda )}{1-\lambda } A$ as in Lemma 4.1. Also here, we have further candidates, but any generator *Q* with $M=\textrm{e}^Q$ must have 0 as an eigenvalue and thus spectrum $\sigma (Q) = \{ 0, 0, \log (\lambda ) \pm 2 \pi \textrm{i}k \}$ for some $k\in \mathbb {N}$, where at most finitely many *k* can give a solution. Since we consider real matrices, we need to work with the matrices of the form , as in Corollary 2.11(1).

Otherwise, when $\lambda <0$, we must have $\lambda > -1$ due to Elfving’s condition (Fact 2.8). In fact, as we shall see, embeddability in this case is impossible if $|\lambda |> \textrm{e}^{-\pi }$. Here, we need Corollary 2.11(2). Putting the pieces together and applying (Casanellas et al. 2023, Lemma 3.1), one gets the following result.

#### Lemma 4.2

Let $M\in \mathcal {M}_4$ have $\deg (q_{_M})=2$ and JNF , with $0 \ne \lambda \in [-1,1)$. Then, $M = T^{-1} \textrm{diag}(1, 1, \lambda , \lambda ) T$ for some $T \in \textrm{GL}(4, \mathbb {R})$. If $\lambda > 0$, *M* is always embeddable, via the generator $Q = - \frac{\log (\lambda )}{1-\lambda } A$ with . Further embeddings exist if and only ifis a generator for some $0\ne k\in \mathbb {Z}$ and some $x,y,z \in \mathbb {R}$ with $yz-x^2=1$ and $z>0$.

Otherwise, if $\lambda < 0$, the matrix *M* is embeddable if and only ifis a generator for some $k\in \mathbb {Z}$ and some $x,y,z \in \mathbb {R}$ with $yz-x^2=1$ and $z>0$.

In both cases, at most finitely many solutions can exist, and the range of *k* can be restricted via $2 \pi |k |\leqslant |\log (\lambda )|$ in the first and via $|2 k + 1|\pi \leqslant \big |\! \log |\lambda |\big |$ in the second case, where the latter excludes $\lambda < - \textrm{e}^{-\pi }$. $\square$

Note that the appearance of the matrices $I_{x,y,z}$ comes from the freedom in choosing *T*. Since $\textrm{diag}(1, 1, \lambda , \lambda )$ certainly commutes with any matrix of the form  with $B\in \textrm{GL}(2,\mathbb {R})$, a proper choice of *B* would mean to simply replace $I_{x,y,z}$ by $I = I^{}_{0,1,1}$. However, since it is not obvious how to achieve this from the start, we need to formulate the result as stated. Once the ‘correct’ *B* is chosen, it becomes transparent why only finitely many candidates exist.

### Cases of degree 3

Taking into account Fact 2.2, there are four possible cases with real spectrum, and one with a complex conjugate pair. First, consider *M* with $\sigma (M)\subset \mathbb {R}$ and JNF $\textrm{diag}(1, 1, \lambda _1, \lambda _2)$, then with $1 \ne \lambda _1 \ne \lambda _2 \ne 1$, which means $\lambda _1, \lambda _2 \in (0,1)$ for embeddability by Fact 2.6. Then, by Lemma 2.12, only one real logarithm with zero row sums exist, and this is , so *M* is embeddable if and only if this is a generator.

Next, let us consider *M* with JNF  and $\lambda \ne 1$, then necessarily with $\lambda \in (0,1)$ for embeddability. Employing (Higham 2008, Thm. 1.27), we see that any real logarithm of *M* must be (complex) similar to $$\textrm{diag}( 2 \pi \textrm{i}k, - 2 \pi \textrm{i}k) \oplus \left( {\begin{matrix} \log (\lambda ) &{} 1/\lambda \\ 0 &{} \log (\lambda ) \end{matrix}} \right) $$ for some $k\in \mathbb {Z}$, where

19 $$\begin{aligned} \exp \begin{pmatrix} \log (\lambda ) &{} 1/\lambda \\ 0 &{} \log (\lambda ) \end{pmatrix} = \begin{pmatrix} \lambda &{} 1 \\ 0 &{} \lambda \end{pmatrix}, \end{aligned}$$

as follows from a simple calculation. Again, only $k=0$ is possible for a generator, in line with Lemma 2.12, and *M* and *Q* then have the same centraliser. This once again means that the principal matrix logarithm gives the only candidate, and we have the following.

#### Lemma 4.3

Let $M\in \mathcal {M}_4$ have minimal polynomial of degree 3 and the JNF , with $1 \ne \lambda _1 \ne \lambda _2 \ne 1$, or JNF , with $\lambda \ne 1$. Then,  is embeddable if and only if the following two conditions are satisfied.

1. One has $\lambda _1, \lambda _2 \in (0,1)$, respectively $\lambda \in (0,1)$, which implies $\varrho _{_{A}} < 1$.
2. The matrix  is a generator.

In this case, the embedding is unique.

Concretely, setting $\lambda _1 = 1+\mu$ and $\lambda _2=1+\nu$ in the first case, and $\lambda = 1+\mu$ in the second, the logarithm can be calculated via the SMT from Eq. (3) aswith the coefficients from Eq. (14) in the first and from Eq. (15) in the second case. $\square$

Next is the case with JNF $\textrm{diag}(1, \lambda ) \oplus \mathbb {J}_2 (\lambda )$ and $\lambda \ne 1$, which also has $\deg (q_{_M})=3$. Again, it can only be embeddable if $\lambda \in (0,1)$ by Fact 2.6. Then, we have $\varrho _{_A} < 1$, and  is well defined and the only real logarithm of *M*. It is similar to $$\textrm{diag}\bigl (0, \log (\lambda )\bigr ) \oplus \left( {\begin{matrix} \log (\lambda ) &{} 1/\lambda \\ 0 &{} \log (\lambda ) \end{matrix}} \right) $$, compare Eq. (19), and we have the following.

#### Lemma 4.4

Let $M\in \mathcal {M}_4$ have JNF $\textrm{diag}(1,\lambda ) \oplus \mathbb {J}_2 (\lambda )$ with $\lambda \ne 1$. Then, $\deg (q_{_M}) = 3$ and *M* is embeddable if and only if the following two conditions are satisfied.

1. One has $\lambda \in (0,1)$, which implies $\varrho _{_{A}} < 1$.
2. The matrix  is a generator.

In this case, the embedding is unique. Further, with $\lambda = 1 + \mu$, one has  with $\alpha$ and $\beta$ as in Eq. (15). $\square$

Note that the matrix *M* here is not cyclic, but we still get a unique real logarithm. This case can also be seen as a limit of the cyclic matrices from Proposition 4.11 below.

For $\sigma (M)\subset \mathbb {R}$, it remains to consider the case that *M* has JNF $\textrm{diag}(1, \lambda _1, \lambda _2, \lambda _2)$ with $1 \ne \lambda _1 \ne \lambda _2 \ne 1$. Embeddability forces $\lambda _1 \in (0,1)$, but $\lambda _2$ can be positive or negative. Let us first look at $\lambda _2 > 0$. Then, *A* has spectral radius  is well defined, but need not be a generator, or even if it is, it need not be the only one. So, we need again the matrices $I_{x,y,z}$ from Fact 2.10 to proceed.

Any generator *Q* with $M=\textrm{e}^Q$ must have spectrum

$$\begin{aligned}\sigma (Q) = \{ 0, \log (\lambda _1), \log (\lambda _2) \pm 2 \pi \textrm{i}k \} \end{aligned}$$

for some $k\in \mathbb {N}_0$, where finitely many $k\ne 0$ can give a solution even if  fails. The case $\lambda _2<0$ is similar, except that  does not converge as a series. Corollary 2.11 gives us the form of the real matrices we need to work with, as for Lemma 4.2, and the two cases $\lambda _2>0$ and $\lambda _2<0$ have to be treated separately as follows, invoking again (Casanellas et al. 2023, Lemma 3.1).

#### Lemma 4.5

Let $M\in \mathcal {M}_4$ have $\deg (q_{_M})=3$ and JNF $\textrm{diag}(1, \lambda _1, \lambda _2, \lambda _2)$, together with $1 \ne \lambda _1 \ne \lambda _2 \ne 1$. Then, $M = T^{-1} \textrm{diag}(1, \lambda _1, \lambda _2, \lambda _2) T$ for some $T \in \textrm{GL}(4, \mathbb {R})$. Now, if all eigenvalues are positive, *M* is embeddable if and only ifis a generator for some $k\in \mathbb {Z}$ and some $x,y,z \in \mathbb {R}$ with $yz-x^2=1$ and $z>0$, where *k* must also satisfy the condition $2 \pi |k |\leqslant |\log (\lambda _2)|$.

Likewise, if $\lambda _1>0$ but $\lambda _2<0$, the matrix *M* is embeddable if and only ifis a generator for some $k\in \mathbb {Z}$ and some $x,y,z \in \mathbb {R}$ with $yz-x^2=1$ and $z>0$. Here, *k* is further restricted via $|2 k + 1 |\pi \leqslant \big |\! \log |\lambda _2|\big |$, which excludes $\lambda _2 < - \textrm{e}^{-\pi }$. $\square$

Once again, by choosing *T* properly, one can replace $I_{x,y,z}$ by $I = I^{}_{0,1,1}$, which makes the structure a little more transparent.

#### Remark 4.6

A special case of Lemma 4.5 occurs when *M* has JNF $\textrm{diag}(1,\lambda ,-\lambda ,-\lambda )$ with $\lambda \in (0,1)$. If such an *M* is embeddable, one has $M=\textrm{e}^Q$ where *Q* must have spectrum $\sigma (Q) = \{0,\mu ,\mu \pm (2k{+}1)\pi \textrm{i}\}$ with $\mu =\log (\lambda )$, for some $k\in \mathbb {Z}$. Then, we get $M^2 = \textrm{e}^{2 Q}$ with JNF $\textrm{diag}(1, \lambda ^2,\lambda ^2,\lambda ^2)$, which means that $M^2$ must be an equal-input matrix; compare the discussion around Lemma 4.1. Since $M^2$ is embeddable by construction, we know that its summatory parameter $c^{\prime } = 1-\lambda ^2$ must lie in (0, 1), as it does, and $M^2$ is also equal-input embeddable. Since *Q* cannot be of equal-input type, we see here one Markov semigroup of the form $\{ \textrm{e}^{t Q}: t \geqslant 0\}$ crossing another, which is the origin of multiple embeddings. $\Diamond$

Finally, we need to look at *M* with JNF $\textrm{diag}(1, 1, \lambda , \overline{\lambda }\, )$ and $\lambda \in \mathbb {C}{\setminus } \mathbb {R}$. As we must have 0 as an eigenvalue of any generator for *M*, we can almost repeat the arguments used for Proposition 3.11, invoke (Casanellas et al. 2023, Thm. 4.5) for $d=4$, and then arrive at the following result,

#### Lemma 4.7

Let the JNF of $M\in \mathcal {M}^{}_{4}$ be $\textrm{diag}(1, 1, \lambda , \overline{\lambda })$, with $\lambda \in \mathbb {C}{{\setminus }} \mathbb {R}$. Then, $\deg (q_{_M})=3$, and *M* is embeddable if and only if the following two conditions are satisfied, with .

1. One has $$0< |\lambda |< 1$$.
2. There is a $k\in \mathbb {Z}$ such that the real logarithm $R^{}_{k} =\alpha ^{}_{k} A + \beta ^{}_{k} A^2$ of *M*, with $\alpha ^{}_{k}$ and $\beta ^{}_{k}$ from Eq. (16), is a generator, which then gives $M=\textrm{e}^{Q}$ with $Q=R^{}_{k}$.

In this case, $R^{}_{k}$ is a generator for only finitely many $k\in \mathbb {Z}$, and no other candidates exist. The number of solutions is bounded by the integer $\big \lfloor 1 - \frac{\log (\det (M))}{2 \pi }\big \rfloor$. $\square$

Let us now turn to the generic situation for $d=4$.

### Cyclic cases

A cyclic matrix $M\in \mathcal {M}^{}_{4}$ can be diagonalisable, in which case it is simple, or not, where it then has a non-trivial JNF. Each case has two subcases to consider. Common to all cases is that any potential generator for the embedding must be a real logarithm of *M* of the form $R = \alpha A + \beta A^2 + \gamma A^3$ with  and $\alpha , \beta , \gamma \in \mathbb {R}$, by Corollary 2.13.

When $M \in \mathcal {M}^{}_{4}$ is simple and potentially embeddable, there are two cases to consider, namely $\sigma (M) = \{ 1, \lambda ^{}_{1}, \lambda ^{}_{2}, \lambda ^{}_{3} \}$ with distinct $\lambda _i \in (0, 1)$, again by Fact 2.6, and otherwise $\sigma (M) = \{ 1, \lambda , \vartheta , \overline{\vartheta } \}$ with $\lambda \in (0,1)$ and $\vartheta \in \mathbb {C}{{\setminus }}\mathbb {R}$ together with $|\vartheta |< 1$, where $|\vartheta |= 1$ is excluded by a result due to Elfving; see Fact 2.8.

If $\sigma (M)\subset \mathbb {R}$, we are back to Theorem 2.5. Indeed, with $\lambda _i = 1 +\mu _i$, the SMT from Eq. (3) gives the candidate $R = \alpha A + \beta A^2 + \gamma A^3$ with

$$\begin{aligned} \begin{pmatrix} \mu ^{}_{1} &{} \mu ^{2}_{1} &{} \mu ^{3}_{1} \\ \mu ^{}_{2} &{} \mu ^{2}_{2} &{} \mu ^{3}_{2} \\ \mu ^{}_{3} &{} \mu ^{2}_{3} &{} \mu ^{3}_{3} \end{pmatrix} \begin{pmatrix} \alpha \\ \beta \\ \gamma \end{pmatrix} = \begin{pmatrix} \log (\lambda ^{}_{1}) \\ \log (\lambda ^{}_{2}) \\ \log (\lambda ^{}_{3}) \end{pmatrix}. \end{aligned}$$

This leads to the unique, real solution

20 $$\begin{aligned} \begin{pmatrix} \alpha \\ \beta \\ \gamma \end{pmatrix} \, = \, \begin{pmatrix} \frac{\mu ^{}_{2} \mu ^{}_{3}}{m^{}_{1}} &{} \frac{\mu ^{}_{1} \mu ^{}_{3}}{m^{}_{2}} &{} \frac{\mu ^{}_{1} \mu ^{}_{2}}{m^{}_{3}} \\ - \frac{\mu ^{}_{2} + \mu ^{}_{3}}{m^{}_{1}} &{} - \frac{\mu ^{}_{1} + \mu ^{}_{3}}{m^{}_{2}} &{} - \frac{\mu ^{}_{1} + \mu ^{}_{2}}{m^{}_{3}} \\ \frac{1}{m^{}_{1}} &{} \frac{1}{m^{}_{2}} &{} \frac{1}{m^{}_{3}} \end{pmatrix} \begin{pmatrix} \log (\lambda ^{}_{1}) \\ \log (\lambda ^{}_{2}) \\ \log (\lambda ^{}_{3}) \end{pmatrix} \end{aligned}$$

with $m_i = \mu _i \prod _{j\ne i} (\mu _{j} - \mu _{i})$. Here, on the right-hand side, one always has to use the real logarithm, as no other choice results in a real solution. So, we have the following result.

#### Proposition 4.8

Let $M\in \mathcal {M}^{}_{4}$ be simple, with real spectrum. Then, *M* is embeddable if and only if the following two conditions are satisfied, where  as before.

1. All eigenvalues of *M* are positive, which automatically implies $\varrho _{_A} <1$.
2. The real logarithm  is a generator, where $R=\alpha A + \beta A^2 + \gamma A^3$ with the coefficients $\alpha , \beta , \gamma$ from Eq. (20).

In this case, the embedding is unique. $\square$

Next, consider the case that $\sigma (M) = \{ 1, \lambda , \vartheta , \overline{\vartheta } \}$, where embeddability forces $\lambda \in (0,1)$ by Culver’s criterion (Fact 2.6). We must also have $|\vartheta |< 1$, again by Fact 2.8, where we may assume $\textrm{Im}(\vartheta ) > 0$. This implies $\sigma (A) = \{ 0, \mu , \nu , \overline{\nu } \}$ with $\mu \in (-1,0)$ and suitable conditions for $\nu$. Since $\vartheta \not \in \mathbb {R}$, the SMT implies the equation

$$\begin{aligned} \begin{pmatrix} \mu &{} \mu ^{2} &{} \mu ^{3} \\ \nu &{} \nu ^{2} &{} \nu ^{3} \\ \overline{\nu } &{} \overline{\nu } ^{2} &{} \overline{\nu } ^{3} \end{pmatrix} \begin{pmatrix} \alpha \\ \beta \\ \gamma \end{pmatrix} = \begin{pmatrix} \log (\lambda ) \\ \log (\vartheta ) + k \, 2 \pi \textrm{i}\\ \log (\overline{\vartheta } ) - k \, 2 \pi \textrm{i}\end{pmatrix} \end{aligned}$$

for some $k\in \mathbb {Z}$, where $\log$ is again the standard branch of the complex logarithm. This is the most general case where $\alpha , \beta , \gamma$ are real. Given *k*, the unique solution is

21 $$\begin{aligned} \begin{pmatrix} \alpha \\ \beta \\ \gamma \end{pmatrix} = \begin{pmatrix} \frac{|\nu |^2}{m^{}_{1}} &{} \frac{\mu \overline{\nu } }{m^{}_{2}} &{} \frac{\mu \nu }{m^{}_{3}} \\ -\frac{\nu +\overline{\nu } }{m^{}_{1}} &{} -\frac{\mu +\overline{\nu } }{m^{}_{2}} &{} -\frac{\mu +\nu }{m^{}_{3}} \\ \frac{1}{m^{}_{1}} &{} \frac{1}{m^{}_{2}} &{} \frac{1}{m^{}_{3}} \end{pmatrix} \begin{pmatrix} \log (\lambda ) \\ \log (\vartheta ) + k \, 2 \pi \textrm{i}\\ \log (\overline{\vartheta } ) - k \, 2 \pi \textrm{i}\end{pmatrix}, \end{aligned}$$

with $m^{}_{1} = \mu (\mu -\nu )(\mu -\overline{\nu } )$, which is real, and $m^{}_{2} = \nu (\nu -\mu )(\nu -\overline{\nu } )$ and $m^{}_{3} = \overline{\nu } (\overline{\nu } -\mu )(\overline{\nu } -\nu )$, which form a complex-conjugate pair. In particular, for each $k\in \mathbb {Z}$, we get precisely *one* real logarithm of *M* this way, which need not be a generator though; bounds on *k* follow again from Casanellas et al. (2023, Thm. 4.5). The remainder of the arguments runs in complete analogy to previous ones, and gives the following result.

#### Proposition 4.9

Let $M\in \mathcal {M}^{}_{4}$ be simple, with one complex-conjugate pair of eigenvalues. Then, *M* is embeddable if and only if the following two conditions are satisfied.

1. One has $\sigma (M) = \{ 1, \lambda , \vartheta , \overline{\vartheta } \}$ with $\lambda \in (0,1)$ and $0<|\vartheta |< 1$.
2. One of the real logarithms of $M$, as given by $R_{k}=\alpha A + \beta A^2 + \gamma A^3$ for some $k\in \mathbb {Z}$ with $\alpha , \beta , \gamma$ from Eq. (21), is a generator.

Here, at most finitely many $k\in \mathbb {Z}$ can lead to a generator, and no further candidates exist. The number of solutions is bounded by the integer $\big \lfloor 1 - \frac{\log (\det (M))}{2 \pi }\big \rfloor$. $\square$

It remains to consider the two cases where *M* is cyclic, but not diagonalisable. First, we have $\sigma (M) = \{ 1, \lambda \}$ with $\lambda \in \mathbb {R}$ and $\lambda \ne 1$, where embeddability forces $\lambda \in (0,1)$ by Fact 2.6, and the JNF of *M* must then be $1 \oplus \mathbb {J}^{}_{3} (\lambda )$ by Fact 2.2. The real logarithm of *M* is unique by Lemma 2.12, and it can easily be calculated as follows. Setting $\lambda = 1 + \mu$, the results from Baake and Sumner (2022, Thm. 5.3), as detailed in its proof, imply the confluent Vandermonde-type condition

$$\begin{aligned} \begin{pmatrix} \mu &{} \mu ^2 &{} \mu ^3 \\ 1 &{} 2 \mu &{} 3 \mu ^2 \\ 0 &{} 2 &{} 6 \mu \end{pmatrix} \begin{pmatrix} \alpha \\ \beta \\ \gamma \end{pmatrix} \, = \, \begin{pmatrix} \log (\lambda ) \\ \lambda ^{-1} \\ - \lambda ^{-2} \end{pmatrix} \end{aligned}$$

with the unique solution

22 $$\begin{aligned} \begin{pmatrix} \alpha \\ \beta \\ \gamma \end{pmatrix} = \begin{pmatrix} \frac{3}{\mu } &{} -2 &{} \frac{\mu }{2} \\ -\frac{3}{\mu ^2} &{} \frac{3}{\mu } &{} -1 \\ \frac{1}{\mu ^3} &{} -\frac{1}{\mu ^2} &{} \frac{1}{2 \mu } \end{pmatrix} \begin{pmatrix} \log (\lambda ) \\ \lambda ^{-1} \\ - \lambda ^{-2} \end{pmatrix}. \end{aligned}$$

This case can be summarised as follows.

#### Proposition 4.10

A cyclic matrix  with JNF $1 \oplus \mathbb {J}^{}_{3} (\lambda )$ is embeddable if and only if the following two conditions are satisfied.

1. One has $$\lambda \in (0,1)$$.
2. The real logarithm  with $\alpha , \beta , \gamma$ from Eq. (22) is a generator.

In this case, the embedding is unique. $\square$

The remaining case is $\sigma (M) = \{ 1, \lambda ^{}_{1}, \lambda ^{}_{2}\} \subset \mathbb {R}$, where $1\ne \lambda ^{}_{1} \ne \lambda ^{}_{2} \ne 1$ and, without loss of generality, *M* has JNF $\textrm{diag}(1, \lambda ^{}_{1}) \oplus \mathbb {J}^{}_{2} (\lambda ^{}_{2})$. By Fact 2.6, embeddability is at most possible for $\lambda ^{}_{1}, \lambda ^{}_{2} \in (0,1)$, and the real logarithm of *M* is unique by Lemma 2.12. Here, the results from Baake and Sumner (2022, Thm. 5.3) give

$$\begin{aligned} \begin{pmatrix} \mu ^{}_{1} &{} \mu ^{2}_{1} &{} \mu ^{3}_{1} \\ \mu ^{}_{2} &{} \mu ^{2}_{2} &{} \mu ^{3}_{2} \\ 1 &{} 2 \mu ^{}_{2} &{} 3 \mu ^{2}_{2} \end{pmatrix} \begin{pmatrix} \alpha \\ \beta \\ \gamma \end{pmatrix} = \begin{pmatrix} \log (\lambda ^{}_{1}) \\ \log (\lambda ^{}_{2})\\ \lambda ^{-1}_{2} \end{pmatrix}, \end{aligned}$$

with the unique solution

23 $$\begin{aligned} \begin{pmatrix} \alpha \\ \beta \\ \gamma \end{pmatrix} = \begin{pmatrix} \frac{\mu ^{2}_{2}}{\mu ^{}_{1} (\mu ^{}_{1} - \mu ^{}_{2})^2} &{} \frac{\mu ^{}_{1} (2 \mu ^{}_{1} - 3 \mu ^{}_{2})}{\mu ^{}_{2} ( \mu ^{}_{1} - \mu ^{}_{2})^2} &{} \frac{-\mu ^{}_{1}}{\mu ^{}_{1} - \mu ^{}_{2}} \\ \frac{-2 \mu ^{}_{2}}{\mu ^{}_{1} (\mu ^{}_{1} - \mu ^{}_{2})^2} &{} \frac{3 \mu ^{2}_{2} - \mu ^{2}_{1}}{\mu ^{2}_{2} ( \mu ^{}_{1} - \mu ^{}_{2})^2} &{} \frac{\mu ^{}_{1} + \mu ^{}_{2}}{\mu ^{}_{2} (\mu ^{}_{1} - \mu ^{}_{2})} \\ \frac{1}{\mu ^{}_{1} (\mu ^{}_{1} - \mu ^{}_{2})^2} &{} \frac{\mu ^{}_{1} - 2 \mu ^{}_{2}}{\mu ^{2}_{2} ( \mu ^{}_{1} - \mu ^{}_{2})^2} &{} \frac{-1}{\mu ^{}_{2} (\mu ^{}_{1} - \mu ^{}_{2})} \end{pmatrix} \begin{pmatrix} \log (\lambda ^{}_{1}) \\ \log (\lambda ^{}_{2})\\ \lambda ^{-1}_{2} \end{pmatrix}. \end{aligned}$$

This implies the following result.

**Table 2:** Summary of embedding cases for $d=4$

$\deg (q_{_M})$	JNF	condition(s)	Unique	Details
1		–	Yes	Fact 2.9
2	$$\begin{array}{l} \textrm{diag}(1,1,1,\lambda ) \\ 1 \ne \lambda \in [-1,1) \end{array}$$	$\lambda \in (0,1)$	$$\begin{array}{l} \text {Not} \\ \text {Always} \end{array}$$	Lemma 4.1
	$$\begin{array}{l} \textrm{diag}(1,\lambda ,\lambda ,\lambda ) \\ 1 \ne \lambda \in [-1,1) \end{array}$$	$\lambda \in (0,1)$	$$\begin{array}{l} \text {Not} \\ \text {Always} \end{array}$$	Lemma 4.1
	$$\begin{array}{l} \textrm{diag}(1,1,\lambda ,\lambda ) \\ \lambda > 0 \end{array}$$	$\lambda \in (0,1)$	$$\begin{array}{l} \text {Not} \\ \text {Always} \end{array}$$	Lemma 4.2
	$$\begin{array}{l} \textrm{diag}(1,1,\lambda ,\lambda ) \\ \lambda < 0 \end{array}$$	$\text {some } R_k (x,y,z) \text { is MG}$	$$\begin{array}{l} \text {Not} \\ \text {Always} \end{array}$$	Lemma 4.2
3	$$\begin{array}{l} \textrm{diag}(1,1,\lambda _1,\lambda _2) \\ 1 \ne \lambda _1 \ne \lambda _2 \ne 1 \end{array}$$		Yes	Lemma 4.3
			Yes	Lemma 4.3
	$$\begin{array}{l} \textrm{diag}(1,\lambda ) \oplus \mathbb {J}_2 (\lambda ) \\ \lambda \ne 1 \end{array}$$		Yes	Lemma 4.4
	$$\begin{array}{l} \textrm{diag}(1,\lambda _1,\lambda _2,\lambda _2) \\ 1 \ne \lambda _1 \ne \lambda _2 \ne 1 \end{array}$$	$$\begin{array}{l} \lambda _1, \lambda _2 \in (0,1) \\ \text {some} R_k (x,y,z) \text {is MG} \end{array}$$	$$\begin{array}{l} \text {Not} \\ \text {Always} \end{array}$$	Lemma 4.5
		$$\begin{array}{l} \lambda _1 \in (0,1), \, \lambda _2 < 0 \\ \text {some} R_k (x,y,z) \text {is MG} \end{array}$$	$$\begin{array}{l} \text {Not} \\ \text {Always} \end{array}$$	Lemma 4.5
	$$\begin{array}{l} \textrm{diag}(1,1,\lambda ,\overline{\lambda }\, ) \\ \lambda \in \mathbb {C}{\setminus } \mathbb {R}\end{array}$$	$$\begin{array}{l} |\lambda |\in (0,1) \\ \text {some} R_k \text {is MG} \end{array}$$	$$\begin{array}{l} \text {Not} \\ \text {Always} \end{array}$$	Lemma 4.7
4	$$\begin{array}{l} \textrm{diag}(1,\lambda _1,\lambda _2,\lambda _3) \\ \text {simple spectrum} \end{array}$$		Yes	Prop. 4.8
	$$\begin{array}{l} \textrm{diag}(1,\lambda ,\vartheta , \overline{\vartheta }) \\ 1 \ne \lambda \in [-1,1), \, \vartheta \in \mathbb {C}{\setminus } \mathbb {R}\end{array}$$	$$ \begin{array}{l} \lambda \in (0,1), \, |\vartheta |\in (0,1) \\ \text {some} R_k \text {is MG} \end{array} $$	$$\begin{array}{l} \text {Not} \\ \text {Always} \end{array}$$	Prop. 4.9
	$$\begin{array}{l} 1 \oplus \mathbb {J}_3 (\lambda ) \\ 1 \ne \lambda \in [-1,1) \end{array} $$		Yes	Prop. 4.10
	$$\begin{array}{l} \textrm{diag}(1,\lambda _1) \oplus \mathbb {J}_2 (\lambda _2) \\ 1 \ne \lambda _1 \ne \lambda _2 \ne 1 \end{array}$$		Yes	Prop. 4.11

#### Proposition 4.11

Let $M\in \mathcal {M}^{}_{4}$ be cyclic with $\sigma (M) = \{ 1, \lambda ^{}_{1}, \lambda ^{}_{2} \}$, in this case for $1 \ne \lambda _1 \ne \lambda _2 \ne 1$, and JNF $\textrm{diag}(1, \lambda ^{}_{1}) \oplus \mathbb {J}^{}_{2} (\lambda ^{}_{2})$. Then,  is embeddable if and only if the following two conditions hold.

1. One has $\lambda ^{}_{1}, \lambda ^{}_{2} \in (0,1)$, with $\lambda ^{}_{1} \ne \lambda ^{}_{2}$.
2. The matrix $\alpha A + \beta A^2 + \gamma A^3$ with $\alpha , \beta , \gamma$ from Eq. (23) is a generator.

In this case, the embedding is unique. $\square$

The basic results of this section are summarised in Table 2. In combination with Casanellas et al. (2023), this can be turned into an algorithmic approach to the embedding problem for $d=4$.

## Application to phylogenetics

In this section, we will briefly discuss some models that are in use for the nucleotide mutation schemes in molecular evolution. We shall always use the ordering (*A*, *G*, *C*, *T*).

First, and perhaps simplest, let us look at the widely used *equal-input* model (Steel 2016; Baake and Sumner 2022). The Markov matrices of equal-input type have the form24where the *C*-matrix contains four equal rows of the form $(c^{}_{1}, \ldots , c^{}_{4} )$, with parameters $c_i \geqslant 0$ and summatory parameter $c = c^{}_{1} + \cdots + c^{}_{4}$. For $M_c$ to be Markov, we also need $c \leqslant 1 + c_i$ for all *i*, which further implies $0 \leqslant c \leqslant \frac{4}{3}$. Since $d=4$ is even, Baake and Sumner (2022, Prop. 2.12) or an application of Lemma 4.1 gives the following consequence.

### Corollary 5.1

The four-dimensional equal-input Markov matrix $M_c$ from Eq. (24) is embeddable if and only if its summatory parameter satisfies $0\leqslant c < 1$. In this case, one embedding is $M_c = \textrm{e}^Q$ with the equal-input generator

$$\begin{aligned} Q = \frac{-\log (1-c)}{c_{}} A , \end{aligned}$$

where  and  for $c=0$. For $c>0$, there can be at most finitely many other embeddings, but none with an equal-input generator. $\square$

This contains the constant-input matrices as the special case $c^{}_{1} = \cdots = c^{}_{4} = \frac{c}{4}$, which comprises the Jukes–Cantor matrices from Jukes and Cantor (1969). Unlike the situation of odd dimension, compare Example 3.7 and Baake and Sumner (2022), no further embeddable case can occur here.

A mild extension of the equal-input class is provided by the *Tamura–Nei* (TN) model from Tamura and Nei (1993); see also Cooper and Sumner (2023). Here, one considers Markov matrices of the form

25 $$\begin{aligned} M = \begin{pmatrix} * &{} a^{}_{2} \kappa ^{}_{1} &{} a^{}_{3} &{} a^{}_{4} \\ a^{}_{1} \kappa ^{}_{1} &{} * &{} a^{}_{3} &{} a^{}_{4} \\ a^{}_{1} &{} a^{}_{2} &{} * &{} a^{}_{4} \kappa ^{}_{2} \\ a^{}_{1} &{} a^{}_{2} &{} a^{}_{3} \kappa ^{}_{2} &{} * \end{pmatrix} \end{aligned}$$

with $a^{}_{i} \geqslant 0$ and $\kappa ^{}_{j}\geqslant 0$ for all *i*, *j*, subject to the condition that *M* is Markov, which means that the sum of the off-diagonal elements in each row must not exceed 1. The $*$ in each row is the unique number to ensure row sum 1. Similarly, when the row sums are all 0, we shall speak of a TN generator. The algebraic structure of TN generators is such that their exponential is always a Markov matrix of TN type, see Cooper and Sumner (2023), while it is not clear that a real logarithm should preserve the structure.

TN matrices occur within the often-used hierarchy of *time-reversible* models, like those implemented in popular computational phylogenetics software such as Darriba et al. (2012). While there is no principal reason to restrict to time-reversible models, this class is pretty versatile and seems general enough while having a number of computational advantages; compare (Felsenstein 1973). Time reversibility also implies that these matrices have real spectrum, and they include the HKY matrices from Hasegawa et al. (1985) via $\kappa ^{}_{1} = \kappa ^{}_{2}$.

*M* from (25) is always diagonalisable, with spectrum $\sigma (M) = \{ 1, \lambda _1, \lambda _2, \lambda _3\} \subset \mathbb {R}$ with

$$\begin{aligned} \lambda ^{}_{1}&= 1 - (a^{}_{1} + a^{}_{2} + a^{}_{3} + a^{}_{4}) , \\ \lambda ^{}_{2}&= 1 - \kappa ^{}_{1} (a^{}_{1} + a^{}_{2}) - (a^{}_{3} + a^{}_{4}) , \\ \lambda ^{}_{3}&= 1 - (a^{}_{1} + a^{}_{2}) - \kappa ^{}_{2} (a^{}_{3} + a^{}_{4}) . \end{aligned}$$

The spectrum is generically simple. However, the Markov condition does not imply that all eigenvalues are positive. The $\lambda _i$ all lie in (0, 1) if and only if

26 $$\begin{aligned} 0&< \min \{ 1, \kappa ^{}_{1} \} (a^{}_{1} + a^{}_{2} ) + \min \{ 1, \kappa ^{}_{2} \} (a^{}_{3} + a^{}_{4} ) \quad \text {and} \nonumber \\&\max \{ 1, \kappa ^{}_{1} \} (a^{}_{1} + a^{}_{2} ) + \max \{ 1, \kappa ^{}_{2} \} (a^{}_{3} + a^{}_{4} ) < 1 , \end{aligned}$$

where the first condition ensures that 1 is a simple eigenvalue. This gives the following generic answer, by an application of Proposition 4.8.

### Corollary 5.2

Let $M\in \mathcal {M}_4$ be a TN matrix as in (25), and assume that it is simple. Then, *M* is embeddable if and only if the conditions in (26) are satisfied. In this case, the embedding is unique, and the generator is of TN type. $\square$

When *M* fails to be simple, the spectrum is still real, but has one or several degeneracies. If $\lambda _1=\lambda _2=\lambda _3$, we must have $\kappa ^{}_{1} =1$ or $a^{}_{1}=a^{}_{2}=0$ together with $\kappa ^{}_{2} =1$ or $a^{}_{3}=a^{}_{4}=0$. In any of these cases, one is back to the equal-input matrices, with $c=a^{}_{1} + \cdots + a^{}_{4}$, which are fully covered by Corollary 5.1.

The remaining degenerate cases can lead to the JNF  with $\lambda \in (0,1)$, for instance via $\kappa ^{}_{1}=a^{}_{3} = a^{}_{4}=0$, which is always embeddable by Lemma 4.2, but possibly not in a unique way. Finally, one can have the JNF  within the HKY class (where $\kappa ^{}_{1} = \kappa ^{}_{2}$), with $1,\lambda ,\lambda '$ distinct, then needing Lemma 4.5. We leave further details to the reader.

The *Kimura* 3 ST model, or K3ST for short, was introduced in Kimura (1981) and comprises all Markov matrices of the form27with parameters $x,y,z \geqslant 0$ subject to the condition $x+y+z\leqslant 1$. In each row of *M*, the $*$ again stands for the unique element that makes the row sum equal to 1, and the definition of the matrices $K_i$ is implicit. Under matrix multiplication, the four mutually commuting matrices  form Klein’s 4-group, with $C_2$ denoting the cyclic group with two elements. The class of K3ST *generators* are the matrices of the form28which thus constitute an Abelian class under matrix multiplication.

Note also that, under the (*A*, *G*, *C*, *T*)-ordering, Kimura’s two parameter model from Kimura (1980), called K2P, is the special case where one takes $y=z$. It was discussed in detail, with various surprising results, in Casanellas et al. (2020). Further, the set of matrices that are both K3ST and equal input are precisely the constant-input matrices.

The matrix *M* in (27) is symmetric, hence always has real spectrum, namely

29 $$\begin{aligned} \sigma (M) = \{ 1, 1-2(x+z), 1-2(y+z), 1-2(x+y) \} = \{ 1, \lambda _1, \lambda _2, \lambda _3 \} , \end{aligned}$$

which is meant as a multi-set if degeneracies occur. Here, $\deg (q_{_M})=1$ only for , while $\deg (q_{_M}) = 2$ occurs for $x=y=z\ne 0$. This gives the constant-input matrices, which are covered by Corollary 5.1. Next, when $\deg (q_{_M}) = 3$, we have $x\ne y = z$, which is the K2P model covered in Casanellas et al. (2020), or a scheme that is equivalent to it via a permutation from $S_4$.

Finally, in the generic case that *M* in (27) is simple, which is true if and only if the non-negative numbers *x*, *y*, *z* are distinct, we are in the situation of Proposition 4.8. In particular, *M* has a real logarithm if and only if all its eigenvalues are positive. There is then only one candidate, , which is a generator (Casanellas et al. 2018) if and only if the three non-unit eigenvalues $\lambda _1, \lambda _2, \lambda _3$ satisfy the three inequalities

30 $$\begin{aligned} \lambda _1 \geqslant \lambda _2 \lambda _3 , \quad \lambda _2 \geqslant \lambda _1 \lambda _3, \quad \lambda _3 \geqslant \lambda _1 \lambda _2 . \end{aligned}$$

This can here be derived from Proposition 4.8, which gives an explicit form of the only possible generator, or from an explicit argument based on the diagonalisation of *M* with the involutory Fourier matrix $$W = \frac{1}{2} \left( {\begin{matrix} 1 &{} 1 \\ 1 &{} -1 \end{matrix}} \right) \otimes \left( {\begin{matrix} 1 &{} 1 \\ 1 &{} -1 \end{matrix}} \right) $$ from the discrete Fourier transform over $C_2 {\times } C_2$; see Casanellas et al. (2018) for a previous derivation and some details. The surprisingly simple set of inequalities emerges from additive conditions on the logarithms of the eigenvalues, for the generator property of , upon exponentiation.

### Corollary 5.3

Let the matrix *M* from (27) be simple, so $\sigma (M) = \{ 1, \lambda _1, \lambda _2, \lambda _3 \}$ with distinct $\lambda _i$, all different from 1. Then, *M* is embeddable if and only if all $\lambda _i > 0$ together with obeying the inequalities in (30). In this case, with the $\lambda _i$ in the order from (29) and then setting

$$\begin{aligned} (s^{}_{1}, s^{}_{2}, s^{}_{3}) :=s^{}_{1} \log (\lambda ^{}_1) + s^{}_{2} \log (\lambda ^{}_2) + s^{}_{3} \log (\lambda ^{}_3) \quad \text {for } s^{}_{i} \in \{ +,-\} , \end{aligned}$$

one has $M=\textrm{e}^Q$ with

$$\begin{aligned} Q = \frac{1}{4} \begin{pmatrix} (+,+,+) &{} (-,+,-) &{} (+,-,-) &{} (-,-,+) \\ (-,+,-) &{} (+,+,+) &{} (-,-,+) &{} (+,-,-) \\ (+,-,-) &{} (-,-,+) &{} (+,+,+) &{} (-,+,-) \\ (-,-,+) &{} (+,-,-) &{} (-,+,-) &{} (+,+,+) \end{pmatrix} \end{aligned}$$

which is a K3ST generator, and the embedding is unique. $\square$

There are of course many more 4-dimensional models used in phylogenetics where the embedding problem is relevant and should be studied. For instance, the strand-symmetric model is considered in Casanellas et al. (2022). One interesting result for this model is that the authors identify an open set of embeddable matrices where the principal matrix logarithm is not a generator.

## Extension to time-inhomogeneous cases

The standard continuous-time Markov chain solves the *ordinary differential equation* (ODE) $\dot{M} = M Q$ with a constant generator (or rate matrix) *Q*; see Amann (1990), Walter (1998) for an introduction to classic ODE theory. More generally, in many real-world applications, one has to admit *time-dependent* generators, *Q*(*t*), which leads to the Cauchy (or initial value) problemIf the generators commute, that is, if  for all $t,s \geqslant 0$, as for instance the K3ST generators from Eq. (28) do, the solution is simply given by

31 $$\begin{aligned} M (t) = \exp \left( { \int _{0}^{t} } \! Q(\tau ) \,\textrm{d}\tau \right) , \end{aligned}$$

and any Markov matrix that arises in such a solution is also embeddable in the classic sense discussed above, because $\int _{0}^{t} Q (\tau ) \,\textrm{d}\tau$ is still a generator. However, this might (and will) change once one also considers families of generators that do not commute.

Let us begin by recalling a standard result from ODE theory; see Amann (1990, Ch. III) for background and further details. Concretely, consider the linear ODE on $\mathbb {R}^d$, with a row vector *x*(*t*) in view of our setting, given by

32 $$\begin{aligned} \dot{x} (t) = x(t) Q (t) \end{aligned}$$

with $Q :[t^{}_0, t] \xrightarrow {\phantom{a} } \textrm{Mat}(d, \mathbb {R})$ continuous, where $t > t^{}_0$ is arbitrary, but fixed. We shall also need the limiting case where we let $t \rightarrow \infty$. If *X*(*t*) denotes the corresponding (left) fundamental system, and setting $t^{}_0 = 0$, it satisfies the Cauchy problem33Invoking the transposed version of the classic *Peano–Baker series* (PBS), see Baake and Schlägel (2011) and references therein, one has the following consequence of the Picard–Lindelöf theorem in conjunction with the standard Picard iteration,^2^ as follows from Baake and Schlägel (2011, Thms. 1 and 2) by matrix transposition.

### Proposition 6.1

The Cauchy problem of Eq. (33) with a continuous matrix function *Q* on $\mathbb {R}_{\geqslant 0}$ has a unique solution. It can be represented by the PBSwith $I^{}_1 (t) = \int _{0}^{t} Q (\tau ) \,\textrm{d}\tau$ and the recursive structure $I_{n+1} (t) = \int _{0}^{t} I_n (\tau ) Q (\tau ) \,\textrm{d}\tau$ for $n \in \mathbb {N}$. In particular, this series is compactly convergent in any standard matrix norm. $\square$

Note that the order under the integral is changed in comparison to Baake and Schlägel (2011), which matches the changed order of matrices in (33) and reflects the standard use of row sum normalisation for Markov matrices in probability theory. As explained in detail in Baake and Schlägel (2011), the solution formula with the PBS reduces to the standard one in Eq. (31) when the *Q*(*t*) commute with one another.

### Remark 6.2

Observe that $I^{}_2$ can be calculated in two different ways, namely

$$\begin{aligned} I^{}_2 (t) = \int _{0}^{t} \int _{0}^{t^{}_2} Q(t^{}_1)Q(t^{}_2) \,\textrm{d}t^{}_1 \,\textrm{d}t^{}_2 = \int _{0}^{t} \int _{t^{}_2}^{t} Q(t^{}_2) Q(t^{}_1) \,\textrm{d}t^{}_1 \,\textrm{d}t^{}_2 , \end{aligned}$$

as follows from changing the order of integration together with a change of variable transformation. Then, $I^{}_2$ can also be written as

$$\begin{aligned} I^{}_2 (t) = \frac{1}{2} \int _{0}^{t} \int _{0}^{t} T [ Q(t^{}_1)Q(t^{}_2)] \,\textrm{d}t^{}_1 \,\textrm{d}t^{}_2 \end{aligned}$$

where *T* denotes *time ordering* according to

$$\begin{aligned} T [ Q(t^{}_1) Q(t^{}_2)] :={\left\{ \begin{array}{ll} Q(t^{}_1) Q(t^{}_2), &{} \text {if}\; t^{}_1 \leqslant t^{}_2, \\ Q(t^{}_2) Q(t^{}_1), &{} \text {otherwise}. \end{array}\right. } \end{aligned}$$

Similarly, if $T[Q(t^{}_1)Q(t^{}_{2}) \cdots Q(t^{}_n)]$ denotes the analogous time-ordered version according to $t^{}_1 \leqslant t^{}_2 \leqslant \cdots \leqslant t^{}_n$, one finds the alternative expression

$$\begin{aligned} I^{}_n (t) = \frac{1}{n!} \int _{0}^{t} \int _{0}^{t} \cdots \int _{0}^{t} T[ Q(t^{}_1) Q(t^{}_{2}) \cdots Q(t^{}_n)] \,\textrm{d}t^{}_1 \,\textrm{d}t^{}_2 \cdots \,\textrm{d}t^{}_n , \end{aligned}$$

which is often used in physics, then usually with the opposite ordering due to the action of the matrices to the right (instead of to the left as above). The PBS is then called the Dyson series, or the *time-ordered exponential*, though its actual calculation (if possible at all) is a lot easier with the recursive formulation stated in Proposition 6.1. $\Diamond$

### Theorem 6.3

Consider the Cauchy problem of Eq. (33) under the assumption that *Q*(*t*) is continuous and a Markov generator for all $t \geqslant 0$. Then, the solution flow $\{ X (t): t \geqslant 0 \}$ consists of Markov matrices only.

### Proof

This follows from standard results of ODE theory, as given in Amann (1990, Sec. 16), so we only sketch the required steps.^3^ Consider Eq. (32). Under the assumption on *Q*, for every *x* on the boundary of the closed cone $(\mathbb {R}_{\geqslant 0})^d$, the vector $x Q (t)$ has a direction that points inside the closed cone, because all off-diagonal elements of *Q*(*t*) are non-negative and $x_i=0$ then implies $(x Q (t))_i \geqslant 0$. This means that all non-negative rescalings of $x Q(t)$ remain in the cone and the flow thus cannot pass the boundary to the outside. By the transposed version of Amann (1990, Thm. 16.9 and Cor. 16.10), which formulate the general result from Amann (1990, Thm. 16.5) for this special case, $(\mathbb {R}_{\geqslant 0})^d$ is forward invariant under the flow. Since this applies to any row of *X*(*t*), with , all entries of *X*(*t*) for $t\geqslant 0$ are non-negative.

Now, the row sums of all $I_n (t)$ in the PBS are zero, as can easily be checked inductively, because all operations stay within the (non-unital) algebra $\mathcal {A}^{(d)}_{0}$ of real matrices with zero row sums. Since the first term of the PBS is , we see that the row sums of *X*(*t*) are always 1, which together with the above establishes the Markov property as claimed. $\square$

The fundamental system *X*(*t*) for (32), which solves the matrix equation (33), is the Markov matrix we are after, called *M*(*t*) from now on. Let us add that $\varvec{1} :=(1, \ldots , 1)^{\textsf{T}}$ is a right eigenvector of  with eigenvalue 1. Since *Q*(*t*) is a generator, we get

$$\begin{aligned} \frac{\,\textrm{d}}{\,\textrm{d}t} \bigl ( M(t) \varvec{1} \bigr ) = \dot{M} (t) \varvec{1} = M(t) Q(t) \varvec{1} = \varvec{0} . \end{aligned}$$

This shows that $\varvec{1}$ is a right eigenvector of *M*(*t*) for *all* $t\geqslant 0$, which is another way to see that the row sums of *M*(*t*) are always 1.

### Corollary 6.4

Let $M (t) = X (t)$ be the solution from Theorem 6.3, with *Q*(*t*) being a Markov generator for all $t \geqslant 0$. Then, one has $0 < \det ( M (t)) \leqslant 1$ for all $t \geqslant 0$.

### Proof

Set $w (t) = \det (M (t))$. By Liouville’s theorem (Amann 1990, Prop. 11.4 and Cor. 11.5), which is sometimes also known as Abel’s identity, we then have $\dot{w} (t) = \textrm{tr}(Q (t)) \, w (t)$ for all $t\geqslant 0$, with $w(0) = 1$, and thus

34 $$\begin{aligned} w (t) =\exp \left( {\int _{0}^{t}} \textrm{tr}(Q (\tau )) \,\textrm{d}\tau \right) . \end{aligned}$$

Since, for all $\tau \geqslant 0$, the diagonal elements of $Q (\tau )$ are non-positive, also $\textrm{tr}(Q(\tau ))$ and hence the argument of the exponential are non-positive, and the claim follows from the properties of the exponential function. $\square$

### Remark 6.5

In general, one needs to go beyond continuous families $\{ Q(t): t \geqslant 0 \}$. In particular, one wants to include piecewise continuous functions, so that a jump from one generator to another is covered. This is achieved by simply replacing the Cauchy problem with the corresponding Volterra integral equation,and using the solution theory accordingly. We skip further details of this standard step, and refer to the literature for details (Amann 1990; Walter 1998). As we shall see later, *Q*(*t*) piecewise constant for $t\geqslant 0$ will essentially be sufficient; see Goodman (1970) or Frydman and Singer (1979) for the full measure-theoretic treatment. $\Diamond$

The result of Theorem 6.3 remains true if *Q*(*t*) is piecewise continuous, as one can then simply use the arguments in the proof for each of the finitely many continuity intervals in time, where the initial condition is always the last Markov matrix from the previous interval. Beyond this, if *Q* is locally Lebesgue-integrable, which means that each of its entries is a locally Lebesgue-integrable function, one can approximate *Q*(*t*) on any given compact time interval by step functions, in the sense of standard Lebesgue theory, and use a limit argument; see Lang (1993, Ch. VI.9) for background.

### Corollary 6.6

If *Q*(*t*) is a locally Lebesgue-integrable function of generators, and *M*(*t*) is a solution of the Volterra integral equation from Remark 6.5, this solution is Markov for all $t\geqslant 0$, with . $\square$

An important observation is that *M*(*t*) is absolutely continuous with bounded entries, so $$M(t) Q(t)$$ is again locally integrable. The same type of observation applies to the iterative definition of the $$I_n (t) $$ in the PBS, which implies that each of these integrals defines an absolutely continuous matrix function, with $$I_n (t) \in \mathcal {A}^{(d)}_{0}$$ for all $$n\in \mathbb {N}$$ and $$t\geqslant 0$$, as one can show by induction. This has the following consequence.

### Corollary 6.7

Let $\mathcal {A}$ be a subalgebra of $\mathcal {A}^{(d)}_{0}\!$, hence closed under addition and matrix multiplication, and assume that *Q*(*t*) defines a locally Lebesgue-integrable matrix function of generators, with $Q (t) \in \mathcal {A}$ for all $t\geqslant 0$. Then, the PBS for *M*(*t*) defines an absolutely continuous function of Markov matrices that satisfy  for all $t\geqslant 0$, where each *A*(*t*) is a rate matrix from $\mathcal {A}$.

### Proof

The first integral, $I^{}_1 (t) = \int _{0}^{t} Q(\tau ) \,\textrm{d}\tau$, is well defined and absolutely continuous, with $I^{}_1 (t) \in \mathcal {A}$ for each $t\geqslant 0$. Then, the integrand for $I^{}_2$ is $I^{}_1 Q$, which is Lebesgue integrable on any interval of type [0, *t*], and $I^{}_2 (t) = \int _{0}^{t} I^{}_1 (\tau )Q(\tau ) \,\textrm{d}\tau$ is again well defined and absolutely continuous. Since each $I^{}_2 (t)$ lies once again in $\mathcal {A}$, we can argue inductively and obtain that , where $A(t) = \sum _{m=1}^{\infty } I_m (t)$ converges and lies in $\mathcal {A}$ for each $t\geqslant 0$.

Thus, we see that each *A*(*t*) has zero row sums. Since *M*(*t*) is Markov, by Corollary 6.6, we can conclude that *A*(*t*) is actually a rate matrix, for any $t\geqslant 0$. $\square$

The significance of this statement is that, even under our time-inhomogeneous scheme, the type of generator within a certain class (as given by a subalgebra of $\mathcal {A}^{(d)}_{0}\!$, say) forces the same type of structure on the Markov matrices, as we have seen for the equal-input matrices discussed in and around Corollary 5.1. This can be seen as some kind of consistency property, which needs the algebraic notion.

### Example 6.8

Consider the two symmetric generators

$$\begin{aligned} Q^{}_{1} = \begin{pmatrix} -1 &{} 1 &{} 0 \\ 1 &{} -1 &{} 0 \\ 0 &{} 0 &{} 0 \end{pmatrix} \quad \text {and} \quad Q^{}_{2} \, = \, \begin{pmatrix} -1 &{} 0 &{} 1 \\ 0 &{} 0 &{} 0 \\ 1 &{} 0 &{} -1 \end{pmatrix}, \end{aligned}$$

which lead to the Markov matrices

$$\begin{aligned} M^{}_{1} (t) = \textrm{e}^{t Q_1} = \begin{pmatrix} 1{-}a &{} a &{} 0 \\ a &{} 1{-}a &{} 0 \\ 0 &{} 0 &{} 1 \end{pmatrix} \quad \text {and} \quad M^{}_{2} (s) = \textrm{e}^{s Q_2} = \begin{pmatrix} 1{-}b &{} 0 &{} b \\ 0 &{} 1 &{} 0 \\ b &{} 0 &{} 1{-}b \end{pmatrix} \end{aligned}$$

with $a=\frac{1}{2} (1 - \textrm{e}^{-2 t})$ and $b = \frac{1}{2} (1 - \textrm{e}^{-2s})$. They are also symmetric, while this is no longer the case for the (doubly stochastic) product,

35 $$\begin{aligned} M^{}_{1} (t) M^{}_{2} (s) = \begin{pmatrix} (1{-}a)(1{-}b) &{} a &{} (1{-}a) b \\ a (1{-}b) &{} 1{-}a &{} ab \\ b &{} 0 &{} 1{-}b \end{pmatrix}. \end{aligned}$$

Indeed, the symmetric generators do *not* form an algebra, but sit inside the doubly stochastic generators, which do, and which thus form the relevant class to consider in this context. $\Diamond$

Note that the matrix in (35) is not embeddable, due to the 0 in the last row, though it is a product of two embeddable ones. Thus, we now extend the notion of embedabbility of Markov matrices as follows.

### Definition 6.9

A Markov matrix $M\in \mathcal {M}_d$ is called embeddable *in the generalised sense*, or g-*embeddable* for short, when it occurs as an *X*(*t*) in the solution of Eq. (33), or in the partner Volterra integral equation from Remark 6.5, with $\{ Q (t): t \geqslant 0 \}$ being a locally Lebesgue-integrable family of Markov generators.

The set of all g-embeddable Markov matrices in *d* dimensions is denoted by $\mathcal {M}^{\textrm{ge}}_{d}$.

The more general version with Lebesgue integrability of *Q*(*t*) will effectively be simplified to a piecewise continuous generator family shortly. Clearly, an embeddable Markov matrix is also g-embeddable, via considering a constant function $Q(t)\equiv Q$. For $d=2$, Corollary 6.4 also gives us the following consequence.

### Corollary 6.10

If a Markov matrix $M \in \mathcal {M}_2$ is g-embeddable, it is also embeddable, and the two notions of embeddability agree for $d=2$, so $\mathcal {M}^{\textrm{e}}_{2} = \mathcal {M}^{\textrm{ge}}_{2}\!$.

### Proof

Let $M \in \mathcal {M}_2$ be embeddable in the generalised sense. When *Q*(*t*) is continuous, by Corollary 6.4, we then know that $\det (M) \in (0,1]$, which means it is embeddable by Kendall’s criterion (Theorem 2.4). The conclusion is easily extended to *Q* locally Lebesgue-integrable, because *w*(*t*) from Eq. (34) still is the determinant of *M*(*t*), which is absolutely continuous, hence $\det (M) \in (0,1]$ also in this case.

The other direction is clear. $\square$

Let us next look at a simpler pair of generators than that of Example 6.8.

### Example 6.11

Consider the two non-commuting elementary (or Poisson) generators

$$\begin{aligned} Q^{}_{1} = \begin{pmatrix} -1 &{} 1 &{} 0 \\ 0 &{} 0 &{} 0 \\ 0 &{} 0 &{} 0 \end{pmatrix} \quad \text {and} \quad Q^{}_{2} = \begin{pmatrix} 0 &{} 0 &{} 0 \\ 0 &{} 0 &{} 0 \\ 1 &{} 0 &{} -1 \end{pmatrix}, \end{aligned}$$

which give the exponentials  for $i \in \{ 1,2\}$. One findswith $a = 1-\textrm{e}^{-t}$ and $b=1-\textrm{e}^{-s}$. When $ab>0$, the matrix $M=M(t,s)$ cannot be embeddable because $M_{31} M_{12} > 0$, but $M_{32}=0$, which violates the transitivity property that is necessary for embeddability; see Norris (2005, Thm. 3.2.1) or Baake and Sumner (2020, Prop. 2.1). By construction, each *M*(*t*, *s*) is g-embeddable though, where the time evolution uses a piecewise constant generator family, first being $Q^{}_{2}$ until time *s*, where it switches to $Q^{}_{1}$ and continues until time $t+s$.

Let us also check this via the results discussed earlier. For $t,s > 0$ with $t\ne s$, the spectrum of *M* is $\{1, 1 {-} a, 1 {-} b \}$, hence simple and positive, wherefore we know from Proposition 3.10 that the only possibility for an embedding of  comes fromwith the coefficients calculated via Eq. (14). Since $A^2 = - a^2 Q^{}_{1} - b^2 Q^{}_{2} + a b Z$ with

$$\begin{aligned} Z = Q^{}_{2} Q^{}_{1} = \begin{pmatrix} 0 &{} 0 &{} 0 \\ 0 &{} 0 &{} 0 \\ -1 &{} 1 &{} 0 \end{pmatrix}, \end{aligned}$$

this gives  with

$$\begin{aligned} \gamma&= \frac{a \log (1-b) - b \log (1-a)}{b-a} \\&= - ab \left( \frac{1}{2} + \frac{1}{3} (a+b) + \frac{1}{4} (a^2 + ab + b^2) + \frac{1}{5} (a^3 + a^2 b + a b^2 + b^3) + \cdots \right) \end{aligned}$$

which is clearly negative for any $0< a\ne b < 1$. This carries over to the case $0<a=b$ by a limiting argument of de l’Hospital type, where we note that *M*(*t*, *t*) for $t>0$ is still cyclic, but not diagonalisable. We thus see that the only real logarithm of *M*(*t*, *s*) for $ts>0$ has a negative entry at the second position in its third row, and is thus not a generator. $\Diamond$

Clearly, the same type of argument as in Example 6.11 can be used for any dimension $d>3$, whence we can summarise our informal discussion as follows.

### Fact 6.12

For any $d\geqslant 3$, there are non-embeddable matrices $M \in \mathcal {M}_d$ that are g-embeddable, so $\mathcal {M}^{\textrm{e}}_{d}\subsetneq \mathcal {M}^{\textrm{ge}}_{d}$ for any $d\geqslant 3$. $\square$

Let us state one algebraic advantage of $\mathcal {M}^{\textrm{ge}}_{d}$ that was noticed and used in Johansen (1973, Thm. 2.7).

### Lemma 6.13

The set $\mathcal {M}^{\textrm{ge}}_{d}$ is a monoid under ordinary matrix multiplication, and it is star-shaped with respect to the singular constant-input matrix

$$\begin{aligned} J_d :=\frac{1}{d} \begin{pmatrix} 1 &{} \cdots &{} 1 \\ \vdots &{} \vdots &{} \vdots \\ 1 &{} \cdots &{} 1 \end{pmatrix} = \frac{1}{d} \, C( 1, \ldots , 1) , \end{aligned}$$

which is an idempotent that lies on the boundary of $\mathcal {M}^{\textrm{ge}}_{d}$.

### Proof

The semigroup property of $\mathcal {M}^{\textrm{ge}}_{d}$ is obvious from the definition, and  clearly is its neutral element.

Now, consider , which is a constant-input matrix and as such certainly embeddable (and hence in $\mathcal {M}^{\textrm{e}}_{d}$) for all $0\leqslant c < 1$. For any fixed $P\in \mathcal {M}^{\textrm{ge}}_{d}$, we know that

$$\begin{aligned} P M_c = (1-c) P + c J_d \end{aligned}$$

lies in $\mathcal {M}^{\textrm{ge}}_{d}$ for every $c\in [0,1)$, where we have used that $P J_d = J_d$ holds because *P* is Markov. So, $\mathcal {M}^{\textrm{ge}}_{d}$ is indeed star-shaped from $J_d$, while all other claims are clear. $\square$

It is possible to say more on special matrix classes, one being the equal-input matrices, where one needs to distinguish even and odd dimensions.

### Lemma 6.14

Let $M\in \mathcal {M}_d$ be an equal-input matrix. If *d* is even, *M* is g-embeddable if and only if it is also embeddable. For *d* odd, the time-inhomogeneous approach with all *Q*(*t*) of equal-input type does not produce new g-embeddable cases.

### Proof

When *d* is even and *M* is g-embeddable, its determinant must lie in (0, 1] by Corollary 6.4. Since *M* is equal input, we have $\det (M) = (1-c)^{d-1}$, which implies $c\in [0,1)$, and *M* is also embeddable, with an equal-input generator, by Baake and Sumner (2022, Prop. 2.12).

When *d* is odd, we know that other types of embeddability are possible for $c>1$, but then not with equal-input generators. If *Q*(*t*) is equal input for all $t\geqslant 0$, the solution of $\dot{M} = MQ$ (respectively of the corresponding Volterra integral equation) will be equal input for all $t\geqslant 0$ if *M*(0) is equal input, as follows from the PBS because the algebra $\mathcal {C}^{(d)} \subsetneq \mathcal {A}^{(d)}_{0}$ generated by the equal-input rate matrices is closed under addition and multiplication, so  with $A(t)\in \mathcal {C}^{(d)}$ for all $t\geqslant 0$; compare Corollary 6.7.

Now, assume that *M*(0) has summatory parameter $c(0) < 1$, which is certainly true if . Then, $c(t) < 1$ for all $t\geqslant 0$ because $c(t)=1$ would mean $\det \bigl ( M(t)\bigr )=0$, which is impossible due to Corollary 6.4. Consequently, each *M*(*t*) will also be equal-input embeddable, again by Baake and Sumner (2022, Prop. 2.12), and no new cases emerge here.

The only remaining case is that *M*(0) is equal input and embeddable, but with $c(0)>1$. Then, by a standard calculation, one sees that *M*(*t*) remains equal input, with

$$\begin{aligned} c(t) = c(0) \exp \left( - \int _{0}^{t} \tilde{c} (\tau ) \,\textrm{d}\tau \right) \end{aligned}$$

with $\tilde{c}(\tau )$ being the summatory parameter of $Q(\tau )$. Clearly, *c*(*t*) is non-increasing, and a minor deformation argument around the approach we used in Remark 3.9 shows that *M*(*t*) remains embeddable if *M*(0) is. $\square$

It is clear that a more systematic analysis of g-embeddability of matrix models will be necessary, which we defer to future work. At this point, we take a look at the most general model, which is based on $\mathcal {A}^{(d)}_{0}$ and admits an approach via Poisson matrices. To describe it, let $E_{ij} \in \textrm{Mat}(d,\mathbb {R})$ be the elementary matrix that has a single 1 in position (*i*, *j*) and $0$s everywhere else. They satisfy the multiplication rule $E_{ij} E_{k\ell } \, = \, \delta _{jk} E_{i\ell }$.

A *Poisson matrix* is any Markov matrix of the form  for some $i\ne j$, then with $a\in [0,1]$. Since $\det (M) = 1-a$, the matrix *M* is singular if and only if $a=1$. Likewise, any matrix of the form $Q = -\alpha E_{ii} + \alpha E_{ij}$ with $i\ne j$ and $\alpha \geqslant 0$ is a *Poisson generator*, where one often restricts to $\alpha > 0$ to exclude the trivial case ; compare Example 6.11. A simple calculation shows that $Q^2 = -\alpha Q$ and thenSo, a Poisson matrix, with parameter *a*, is embeddable if and only if $0\leqslant a < 1$, which are precisely all non-singular cases.

### Example 6.15

Let $d=2$ and consider the product of two Poisson matrices,

$$\begin{aligned} \begin{pmatrix} 1{-}a &{} a \\ 0 &{} 1 \end{pmatrix} \begin{pmatrix} 1 &{} 0 \\ b &{} 1{-}b \end{pmatrix} = \begin{pmatrix} 1 {-} a(1{-}b) &{} a(1{-}b) \\ b &{} 1{-}b \end{pmatrix} \end{aligned}$$

for $a,b \in [0,1)$. Both are regular, and so is their product, then clearly with determinant $1- (a+b-ab)$. One can check that $0 \leqslant a+b-ab < 1$, which shows embeddability of the product by Kendall’s criterion from Theorem 2.4. Since $a+b-ab$ can take all values in [0, 1), forming the product of just two Poisson matrices covers all embeddable cases, in line with Corollary 6.10. $\Diamond$

Let us collect some general properties of g-embeddable matrices, starting with the following necessary criterion from Goodman (1970), Frydman (1980a).

### Lemma 6.16

If $M\in \mathcal {M}_d$ is g-embeddable, it must satisfy

$$\begin{aligned} \prod _{i=1}^{d} m^{}_{ii} \geqslant \det (M) > 0 . \end{aligned}$$

So, if $m^{}_{ii} = 0$ for some *i*, the matrix *M* is neither embeddable nor g-embeddable. $\square$

An astonishing general result is the following characterisation of $\mathcal {M}^{\textrm{ge}}_{d}$ due to Johansen.

### Theorem 6.17

(Johansen 1973, Thms. 2.5 and 2.6) Every $M\in \mathcal {M}^{\textrm{ge}}_{d}$ can be approximated arbitrarily well by finite products of Poisson matrices, and every $M\in \textrm{int} (\mathcal {M}^{\textrm{ge}}_{d})$ has a representation as a finite product of Poisson matrices, known as a Bang–Bang representation.

Moreover, Markov idempotents and products of Markov idempotents lie on $\partial \mathcal {M}^{\textrm{ge}}_{d}$. They are finite products of regular or singular Poisson matrices. $\square$

The difficult matrices are thus the non-singular elements on the boundary of $\mathcal {M}^{\textrm{ge}}_{d}$, and no general characterisation seems to be known. The key point of this result is that it is essentially sufficient to consider the ODE from Eq. (33) with families of Markov generators that are piecewise constant, at least in an approximate sense.

### Characterisations for $d = 3$

The situation is a little better here. First, Lemma 6.16 has some partial converse as follows.

#### Proposition 6.18

(Frydman 1980a, Thm. 3.1) Let $M\in \mathcal {M}_3$ satisfy $m^{}_{ij}=0$ for some $i\ne j$. Then, *M* is g-embeddable if and only if the double inequality of Lemma 6.16 holds. These matrices are precisely the ones that can be represented as products of at most 5 Poisson matrices. $\square$

All remaining cases share the condition to be totally positive, so $m^{}_{ij} > 0$ for all $1\leqslant i,j \leqslant 3$. To proceed, we need one further quantity that seems to be specific to $d=3$, and no analogue in higher dimensions has been found so far. Let $M^{(ij)}$ denote the minors of $M$, meaning the determinants of the sub-matrices of *M* that emerge from removing row *i* and column *j*. Then, define the real number

$$\begin{aligned} B_{_M} :=\max _{1\leqslant i,j \leqslant 3} \frac{m^{}_{ii} m^{}_{jj}}{m^{}_{ij}} (-1)^{i+j+\delta _{ij} -1} M^{(ij)}. \end{aligned}$$

Now, we can recall the following results from the literature.

#### Theorem 6.19

If $M\in \mathcal {M}_3$ is totally positive, one has the following results.

1. If $B_{_M} \geqslant \det (M) >0$, the matrix *M* is g-embeddable, and has a representation as a product of at most 6 Poisson matrices.
2. If $\det (M)>0$ with $B_{_M} < \det (M)$, a representation as a product of at most 6 Poisson matrices exists if and only if a set of 9 inequalities and 6 equations is satisfied, as detailed in Frydman (1983, Eq. 1.2).
3. If *M* is g-embeddable, it can always be written as a product of finitely many Poisson matrices, the number of which is bounded by
4. If *M* is g-embeddable with $\det (M) \geqslant \frac{1}{8}$, one has $B_{_M} \geqslant \det (M)$ and a representation as in (1), with at most 6 Poisson matrices.
5. If *M* is g-embeddable with $B_{_M} < \det (M)$, one has a representation as a product of finitely many Poisson matrices. For $k\geqslant 2$, their number is bounded by $n^{}_{k}$, where
  $$\begin{aligned} n^{}_{k} = {\left\{ \begin{array}{ll} 5k-2 , &{} \text {if}\; \tfrac{1}{2\cdot 8^{k-1}_{}} \leqslant \det (M)< \tfrac{1}{8^{k-1}} , \\ 5k-1 , &{} \text {if} \; \tfrac{1^{}}{8^k} \leqslant \det (M) < \tfrac{1}{2\cdot 8^{k-1}} , \end{array}\right. } \end{aligned}$$
  and it is known that 6 is generally not sufficient.

#### Proof

The first two claims follow from Frydman (1983, Thm. 1.1), but are also contained in the earlier work of Frydman (1980a, 1980b).

Claim (3) follows from Johansen and Ramsey (1979, Thm. 10), while Claims (4) and (5) are a mild reformulation of Frydman (1983, Thm. 4.1 and Thm. 4.2), respectively. $\square$

While it is not known how good the representation bounds are with growing *k*, it is clear that the number of matrices in a Bang-Bang representation becomes more difficult to control as the determinant of *M* shrinks.

## Outlook

The classic embedding problem for $d\leqslant 4$ is in fairly good shape, and further classes of examples can be studied or refined with the above tools and criteria. In this context, for $d=4$, the role of algebraic models will become important, as is visible with symmetric versus doubly stochastic generators; compare Example 6.8. Various further classes for $d=4$ are worth looking at, and a revised systematic treatment of embeddability for time-reversible classes of Markov matrices seems useful, too.

While results in more than four dimensions are still limited, they are nonetheless important, also in the context of population genetics. Here, we only mention recombination, which can be understood as a Markov process (Baake and Baake 2016). Clearly, embeddability is an important feature again, and some progress will be possible because, in a natural formulation with posets and lattices, one can work with triangular Markov matrices, where the diagonal elements are the eigenvalues, which then are non-negative real numbers.

Clearly, perhaps the most important next step will be the development of the generalised embedding for $d=4$, as time-inhomogeneous Markov chains are practically important and unavoidable. While we already know that we cannot get all theoretically possible determinants this way, relevant extensions do occur, though many questions remain open. This is even true in the context of equal-input matrices, where one needs to relate products with the natural grading, and to understand precisely which additional cases emerge in this general scheme.

## Data Availability

No data of any kind were produced or processed for this work.

## Footnotes

1 Later on, we refer to this property as equal-input embeddable, and similarly for other model classes.

2 We refer to the WikipediA entry on the Picard–Lindelöf theorem for a summary, and to Walter (1998) for details.

3 To better appreciate the flow property of the solution, one should write *X*(0, *t*) instead of *X*(*t*), and more generally $X(t^{}_{0},t)$ for the solution on the time interval $[t^{}_{0}, t]$ with the fitting initial condition at $t^{}_{0}$, which then leads to the concatenation rule $X(t^{}_{0}, t^{}_{1} ) X(t^{}_{1}, t)= X(t^{}_{0}, t)$; see Amann (1990) for more details.
